# Local angiotensin II contributes to tumor resistance to checkpoint immunotherapy

**DOI:** 10.1186/s40425-018-0401-3

**Published:** 2018-09-12

**Authors:** Guozhu Xie, Tan Cheng, Jie Lin, Lanfang Zhang, Jieling Zheng, Ying Liu, Guobo Xie, Baiyao Wang, Yawei Yuan

**Affiliations:** 1 Department of Radiation Oncology, Nanfang Hospital, Southern Medical University/The First School of Clinical Medicine, Southern Medical University, Guangzhou, 510515 Guangdong China; 2 Department of Radiation Oncology, Affiliated Cancer Hospital & Institute of Guangzhou Medical University, Guangzhou, 510095 Guangdong China; 3 Department of Hepatopancreas Surgery, the First People’s Hospital of Foshan, Foshan, 528000 Guangdong China; 4 School of Computer Science and Technology, Guangdong University of Technology, Guangzhou, 510006 China

**Keywords:** Renin-angiotensin system, Angiotensin II, Checkpoint inhibitor, Immunotherapy, Resistance

## Abstract

**Background:**

Current checkpoint immunotherapy has shown potential to control cancer by restoring or activating the immune system. Nevertheless, multiple mechanisms are involved in immunotherapy resistance which limits the clinical benefit of checkpoint inhibitors. An immunosuppressive microenvironment is an important factor mediating the original resistance of tumors to immunotherapy. A previous report by our group has demonstrated that local angiotensin II (AngII) predominantly exists in a tumor hypoxic microenvironment where hypoxic tumour cells produced AngII by a hypoxia-lactate-chymase-dependent mechanism.

**Results:**

Here, using 4T1 and CT26 syngeneic mouse tumor models, we found that local AngII in the tumor microenvironment was involved in immune escape of tumour cells and an AngII signaling blockage sensitized tumours to checkpoint immunotherapy. Furthermore, an AngII signaling blockage reversed the tumor immunosuppressive microenvironment, and inhibition of angiotensinogen (AGT, a precursor of AngII) expression strongly triggered an immune-activating cytokine profile in hypoxic mouse cancer cells. More importantly, AGT silencing combined with a checkpoint blockage generated an abscopal effect in resistant tumors.

**Conclusion:**

Our study demonstrated an important role of local AngII in the formation of a tumor immunosuppressive microenvironment and its blockage may enhance tumor sensitivity to checkpoint immunotherapy. The combination of an AngII signaling blocker and an immune-checkpoint blockage could be a promising strategy to improve tumors responses to current checkpoint immunotherapy.

**Electronic supplementary material:**

The online version of this article (10.1186/s40425-018-0401-3) contains supplementary material, which is available to authorized users.

## Background

Recently, immunotherapy has shown its potential to control cancer by restoring or activating the immune system. Immune checkpoint antibodies against cytotoxic-T-lymphocyte-associated protein 4 (CTLA-4) or programmed cell death protein 1 (PD-1) have been demonstrated to be effective therapeutic approaches in a variety of cancers [1]. Nevertheless, only a subset of patients exhibit durable responses and nearly 70% of patients cannot generate any response to a single-agent strategies for most kinds of tumors [2]. There are multiple mechanisms involved in immune resistance to these therapies [3–5]. The most important factor allowing for tumor cell immune escape in vivo is the intratumor immunosuppressive microenvironment [6].

The renin-angiotensin system (RAS) is classically known as a circulating or hormonal system that regulates blood pressure, electrolytes, and fluid homeostasis [7]. The classical RAS consists of several components: hepatic-derived precursor angiotensinogen (AGT), renally synthesized renin, pulmonary-bound angiotensin-converting enzyme (ACE), and the physiologically active peptide angiotensin II (AngII). AngII is a key bioactive RAS peptide which elicits its effects by binding to its receptors, AngII type 1 receptor (AT1R) and AngII type 2 receptor (AT2R); most of its effector functions are, however, mediated by AT1R [8]. In addition to this systemic RAS, AngII can also be produced in alternative pathways in local tissues across diverse organ systems including the kidney, heart, vasculature, pancreas, and adipose tissue [9]. AngII overproduction plays an important role in the development of chronic diseases, including atherosclerosis [10], diabetic nephropathy [11], and retinopathy [12].

Interestingly, a retrospective study provided evidence that patients with hypertension taking ACE inhibitors and AngII type 1 receptor blockers (ARBs) had a decreased risk of developing some types of cancers [13]. Recent clinical trials showed the candesartan, one of the ARBs, prolonged patients’ survival and improved clinical outcomes in patients with advanced pancreatic cancer [14, 15]. Sub-clinical studies from animal xenograft models and human malignancies observed the frequent dysregulation of RAS components in the tumor microenvironment and a correlation with disease outcomes [7], indicating an important role for local RAS in modulating tumor biology. Our group has previously reported [16] that in human breast cancer and nasopharyngeal carcinoma, the local AngII predominantly exists in the hypoxic regions of the tumors. We demonstrated that tumor cells in hypoxic microenvironments produced AngII by a hypoxia-lactate-chymase-dependent mechanism, which is different from the classical AGT-renin-ACE-AngII pathway [7].

Other previous studies showed that AngII induced a variety of chronic organ diseases via the infiltration and activation of fibroblasts [17, 18] and macrophages [19, 20]. It is notable that fibroblasts and macrophages are important stromal cells and their activation within the tumor microenvironment, in which they become cancer-associated fibroblasts (CAFs) and tumor-associated macrophages (TAMs), contributes to the formation of an intratumoral immunosuppressive microenvironment [6].

In general, macrophages can be polarized to M1 or M2 phenotype [21]. M1-polarized macrophages, also known as classically activated macrophages, produce pro-inflammatory and immunostimulatory responses to infection. TAMs are thought to more closely resemble M2-polarized macrophages [22], which are activated by Th2 cytokines (e.g., IL-4, IL-10, and IL-13). TAMs are the major immunoregulatory cells and result in the immune suppression in the tumor microenvironment [23]. In breast tumors, macrophages is the most prevalent immune cells, exerting a profound influence over the immunologic state of neoplastic tissues [24]. Recruitment of monocytes and cultivation of TAMs in the tumor microenvironment are now recognized as key features of breast cancer progression [24]. Additionally, recent studies provide strong evidence that TAMs facilitate colorectal cancer growth by altering extracellular matrix remodeling, tumor metabolism, angiogenesis, as well as the tumor microenvironment [25].

Since local AngII is present in the tumor microenvironment [16], there is a possibility that it may mediate the recruitment and activation of fibroblasts and macrophages in the tumor microenvironment to participate in the formation of this immunosuppressive microenvironment.

The 4T1 breast cancer and CT26 colon cancer cells are the most popular syngeneic mouse tumor models, particularly for cancer immune research. More importantly, these two tumor models exhibit different sensitivity to immune checkpoint blocker, in which 4T1 breast tumors are resistant to checkpoint immunotherapy and CT26 colon tumors show modest sensitivity to checkpoint immunotherapy [26, 27]. Here using these two tumor models, we demonstrate that local AngII plays an important role in the formation of a tumor immunosuppressive microenvironment. Blockage of AngII effector functions greatly improves tumor sensitivity to checkpoint immunotherapy.

## Methods

### Reagents

The following antibodies were used for the animal experiments: mCD152 (mCTLA-4) monoclonal antibody (9H10; Bio X Cell), mPD-1 monoclonal antibody (RMP1-14; Bio X Cell), anti-mouse CD8 monoclonal antibody (116-13.1, Bio X Cell), polyclonal Syrian hamster IgG (Isotype Controls of anti-mouse CTLA-4, Bio X Cell ), rat IgG2a isotype control (Isotype Controls of anti-mouse PD-1, Bio X Cell ), and mouse IgG2a isotype control (Isotype Controls of anti-mouse CD8, Bio X Cell).

### Cell lines and culture

4T1 (murine breast cancer cells) and CT26 (murine colorectal adenocarcinoma) were purchased from American Type Culture Collection (ATCC). Both tumor cell lines were grown in RPMI-1640 medium supplemented with 10% fetal bovine serum at 37°C, 5% CO_2_. For hypoxic culture, cells were cultured in a hypoxia chamber (37°C with an atmosphere of 1% O_2_ and 5% CO_2_, balanced with N_2_, and humidified). For normoxic culture, cells were cultured in an atmosphere containing 21% O_2_ (37°C and an atmosphere of 5% CO_2_ equilibrated with atmospheric O_2_ in a humidified incubator).

### Construction of shRNA lentiviral vector and establishment of AGT-silenced cells

The designed shRNA construct, as previous studies [16], contained a unique 19-nt double-stranded AGT target sequence that presented as an inverted complementary repeat, a loop sequence (5’-CTCGAG-3’), the RNA PloIII terminator (5’-TTTTTT-3’), and 5’ single-stranded overhangs for ligation into AgeI- and EcoRI-digested Pglv-u6-Puro lentivirus vector (GenePharma, Shanghai, China). The recombinant vector was named pGLV–AGT–shRNA. The negative control vector (pGLV–NC–shRNA) contained a nonsense shRNA insert in order to control any effects caused by non-RNAi mechanisms. We co-transfected the 293 T cells with three optimized packaging plasmids (pGag/Poll, pRev and pVSV-G) and the pGLV-AGT-shRNA or Pglv-NC-shRNA expression clone construct, which produced lentiviral stocks with a suitable titer. Stably transduced 4T1 and CT26 cells were selected using puromycin, adding the minimum concentration of puromycin required to kill untransduced 4T1 or CT26 cells. The efficiency of knockdown was detected by real-time qPCR and western blotting.

### Quantitative RT-PCR

Total RNA was extracted from cells using TRIzol reagent (Invitrogen) according to the manufacturer's protocol. Then, reverse transcription was performed with the PrimeScript® RT reagent kit (Takara), followed by real-time PCR using an ABI 7500 Sequence Detection System with a SYBR® Premix Ex Taq™ kit (Takara). The sequences of the specific PCR primers were as follows:

AGT: Forward: 5′-TGAAGGATACACAGAAGCAA-3′

Reverse: 5′-TGGTAAAGGAGATGGAAGG-3′

GAPDH: Forward: 5′- TGTCGTGGAGTCTACTGGTG-3′

Reverse: 5′-GCATTGCTGACAATCTTGAG-3′

GAPDH was used as the internal control.

### Western blotting

Whole cell lysates were harvested and Western blotting was conducted as described previously [16]. Primary antibodies are listed in Table 1. Blots were visualized using an ECL detection kit (Millipore). Antibodies for Western blotting were listed in Additional file 1: Table S1.

**Table 1:** Cytokines influenced (alteration>2folds) by AGT silence

Cytokines	Functions
Immune-activating	
IL-7	Necessary for both B-cell and T-cell proliferation [43].
IL-20	Enhancing innate and adaptive immunity [44].
CD40 ligand	A potent dendritic cell activation molecule, counteracting immune escape mechanisms in the tumor microenvironment [45, 46].
CXCL1	Pro-inflammatory cytokine: the activation and regulation of innate and adaptive immunity [53].
CXCL11	Chemotactic for activated T cells [47].
CXCL14	Attraction of dendritic cells [48]
TNFSF14	Stimulating lymphocyte proliferation and tumor cell-specific anti-tumor immune responses [49].
Immunosuppressive	
IL-3	Promoting dendritic cells secreting significantly less IL-12 p70 and more IL-10 [54]
IL-4	Participating in both TAM and MDSC survival and the acquisition of an immune-suppressive phenotype [55, 56].
IL-10	Inhibiting the ability of APCs to present antigens to T cells [57]
Fas	Inducing apoptosis of cytotoxic T lymphocytes through the FAS-FASL pathway [58]
Fas ligand (FASL)	Inducing apoptosis of cytotoxic T lymphocytes through the FAS-FASL pathway [58]
CCL1	Specific recruitment of regulatory T cells in ovarian carcinoma fosters immune privilege [59]
CCL7	Chemoattracting MDSCs and Tregs in tumor microenvironment [60].
SDF-1	Increasing immunological tolerance by polarizing Tregs [61].
CCL28	Recruiting Tregs in tumor hypoxia microenvironment [62].
G-CSF	Recruiting MDSCs in tumor hypoxia microenvironment [63].
GM-CSF	Shaping the tumour microenvironment by promoting myelopoiesis and recruitment of suppressive myeloid cells [55, 64, 65]
Eotaxin-2	the recruitment and polarization of Tregs [66, 67].
TNFSF12	Curtailing the innate response and its transition to adaptive TH1 Immunity [68].

### Detection of cell proliferation ability

Cell proliferation ability was detected by MTT assays. Cells were plated into 96-well plates (2×10^3^ cells/well) in 200 μL of medium. After incubation for 8 h, cells were exposed to candesartan (10μm/well, a selective AT1R blocker), PD123319 (10μm/well, a selective AT2R blocker), a combination of these (10μm/well candesartan and 10μm/well PD123319), and control DMSO for 0, 24, 48, 72, and 96 h. At each time point, 20 μL of 5 mg/mL MTT solution in PBS was added to each well for 4 h at 37 °C. Subsequently, a 20% sodium dodecyl sulfate (SDS) solution in 0.01% HCl (150 μL) was added to each well, and the absorbance at 570 nm was measured using a spectrophotometric plate reader (ND-1000, Wilmington, DE). Two independent experiments were performed, each in triplicate.

### Sirius Red staining

Sirius Red staining was performed as previously described [28]. Formalin-fixed tissues were embedded in paraffin and sectioned at 5 μm thickness.Sirius Red staining for collagen was performed using 0.1% Sirius Red (Direct Red80; Sigma) and counterstained with Weigert’s hematoxylin.

### Flow cytometry

The following anti-mouse antibodies were used for flow cytometry: Live/Dead Fixable Near IR Dead Cell Stain (Life Technologies), CD45-PerCP-Cy5.5 (BD Biosciences), CD3-PE (BD Biosciences), CD44-PE (BD Biosciences), CD8a-APC (BD Biosciences), CD4-APC (BD Biosciences), Foxp3-PE (BD Biosciences), CD11b-PE-Cy7 (BD Biosciences), F4/80-APC (BD Biosciences), Ly6C-PE (BD Biosciences), Ly6G-FITC (BD Biosciences), and Ly6G-APC-Cy7 (BD Biosciences). Flow cytometry was performed with Canto II (BD) and the data were analyzed with FlowJo 7.6 software (TreeStar). Live/dead cell discrimination was performed using Live/Dead Fixable Aqua Dead Cell Stain Kit (Life Technologies). All of cell surface or intracellular stainings were done according to the manufacturer's instructions. T effector cells (T_eff_) were phenotyped as CD45^+^CD8^+^CD44^+^, regulatory T cells (T_regs_) as CD45^+^CD4^+^Foxp3^+^, macrophages as CD45^+^CD11b^+^F4/80^+^, tumor-associated macrophages (TAMs) as CD45^+^CD11b^+^F4/80^+^CD206^+^, monocytic myeloid-derived suppressor cells (Mo-MDSCs) as CD45^+^CD11b^+^Ly6G^low^Ly6C^high^, and granulocytic myeloid-derived suppressor cells (G-MDSCs) as CD45^+^CD11b^+^Ly6G^high^Ly6C^low^.

### ELISA

AngII, IL-10, GM-CSF, G-CSF, Eotaxin-2, and CXCL11 ELISA kits were all obtained from Ray Biotech, Inc. (Norcross, GA, USA). TNFSF14 ELISA kits was obtained from CUSABIO Inc. (Wuhan, China). The ELISA for them was carried out according to the manufacturer’s instructions. Two independent experiments were performed, each in triplicate. For AngII ELISA, the microplate in the kit, which is pre-coated with anti-rabbit secondary antibody, was incubated with an anti-AngII antibody, under such conditions that both biotinylated AngII peptide and a peptide standard or targeted peptide in the samples interacted competitively with the AngII antibody. Unbound biotinylated AngII peptide was then allowed to interact with streptavidin-horseradish peroxidase (SA-HRP), which catalyzes a color development reaction. The intensity of the colorimetric signal is directly proportional to the amount of the biotinylated peptide-SA-HRP complex and inversely proportional to the amount of the AngII peptide in the standard or samples. A standard curve of known concentration of AngII peptide was established, and the concentration of AngII peptide in the samples was then calculated by interpolation onto the standard curve. All groups of tumor cells were all seeded at a density of 200,000 cells/well in 1 ml medium in 24-well plates.

### Immunofluorescence

Initially, 5-μm frozen tissue serial sections were fixed with cold acetone for 15 minutes at 4°C and then washed three times for five minutes each with phosphate-buffered saline. Slides were blocked for one hour in 5% bovine serum albumin at room temperature. Primary antibodies were incubated overnight at 4°C. Following additional washes, the slides were incubated for one hour at room temperature with the appropriate secondary antibody and 4',6-diamidino-2-phenylindole (DAPI) counterstaining. To reduce autofluorescence, the slides were subsequently incubated in 0.3 M glycine for 10 minutes and then mounted in Hydromount aqueous mounting medium (Fisher Scientific). Images were acquired with a fluorescence microscope (Olympus BX51). Antibodies used for the immunofluorescence are listed in Additional file 1: Table S1.

### Mouse cytokine antibody array analysis

Cytokine profiles (containing 308 cytokines) of 4T1 cells in normoxic or hypoxic conditions were analyzed using the RayBio Mouse Cytokine Antibody Array. Protein extraction: total protein was extracted from the cells with ice-cold Cell & Tissue Protein Extraction Reagent (KangChen. Cat. # KC-415, China), which contains inhibitors for protein degradation (5ul Protease Inhibitor Cocktail, 5μl PMSF and 5μl Phosphotase Cocktail were added into the 1 ml Protein Extraction Reagent). Determining the protein concentration: the protein concentration was determined by using a BCA Protein Assay Kit (KangChen KC-430, China). Blocking and incubation: a. Protein array membranes were blocked in blocking buffer for 30 min and then incubated with samples at room temperature for 1 to 2h (or incubated at 40 °C overnight). b. Samples were then decanted, and the membranes were washed with washing buffer. After that, the membranes were incubated with diluted biotin-conjugated antibodies at room temperature for 1–2 h. Chemiluminescent detection: the membranes were washed with washing buffer and then reacted with HRP-conjugated streptavidin (1:1000 dilution) at room temperature for 2 h. The membranes were then washed thoroughly and exposed to detection buffer in the dark before being exposed to X-ray film. Afterwards, the membranes were exposed to X-ray film and the image was developed using a film scanner. Data analysis: by comparing the signal intensities, relative expression levels of cytokines were obtained. The intensities of the signals were quantified by densitometry. Positive controls were used to normalize the results from different membranes being compared. Fold changes in protein expression were calculated.

### Animal Models

All experimental procedures were approved and overseen by Southern Medical University Institutional Animal Care and Use Committee, and were performed in accordance with the guidelines and regulations for animal experiments set down by Southern Medical University. Four-week-old female BALB/c mice, BALB/c nude mice and NOD/SCID mice were used for the different animal experiments. 4T1 cells (5 × 10^6^ cells) with or without AGT silencing via AGT shRNA lentiviral transduction were inoculated into the mammary fat pads of each mouse. CT26 cells (5 × 10^6^ cells) with or without AGT silencing were inoculated s.c. into the flank of each mouse. When the tumors were allowed to grow for the indicated time, different drugs or control agents were administered by intraperitoneal injection. Antibodies used for the in vivo immune checkpoint blockade experiments were given intraperitoneally at a dose of 250 μg/mouse every three days (four times total) and included: anti-CTLA4 (9H10), anti-PD-1 (RMP1-14), polyclonal Syrian hamster IgG, and rat IgG2a isotype control. Mice CD8-depletion in vivo was performed according to a previous study [29]. In brief, anti-CD8 antibody or an IgG2a isotype control at a dose of 200 μg/mouse was given 2 days prior to tumor implantations (day −2), day 0, and then every 4 days for the duration of the experiment. To explore the influence of Ang II blockers on tumor growth in vivo, candesartan (5mg/kg, i.p., Takeda Pharmaceutical Company Limited, Japan) and PD123319 (5mg/kg, i.p., Selleck Chemicals) were given every other day starting from day 5 after cell injection. Perpendicular tumor diameters were measured using calipers. Tumor volume was calculated using the formula L × W^2^ × 0.5, where L is the longest diameter and W is the shortest diameter.

### Statistics

Statistical analyses of AngII, GM-CSF and G-CSF levels by ELISA assay, the percentages of Teffs, Tregs, TAMs and MDSCs by Flow cytometry and Teffs / Tregs ratio were performed by using Student’s t test with SPSS 13.0 software. Two-way cell growth in vitro and tumor growth in vivo were evaluated using SPSS 13.0 software with two-way repeated-measures ANOVA. Analyses of survival patterns in tumor-bearing mice were performed using the Kaplan-Meier method, and statistical differences were evaluated according to the Mantel-Cox log-rank test. A *p* value < 0.05 was considered statistically significant.

## Results

### Local AngII in tumor microenvironments is involved in immune escape of tumor cells

**Fig. 1:**
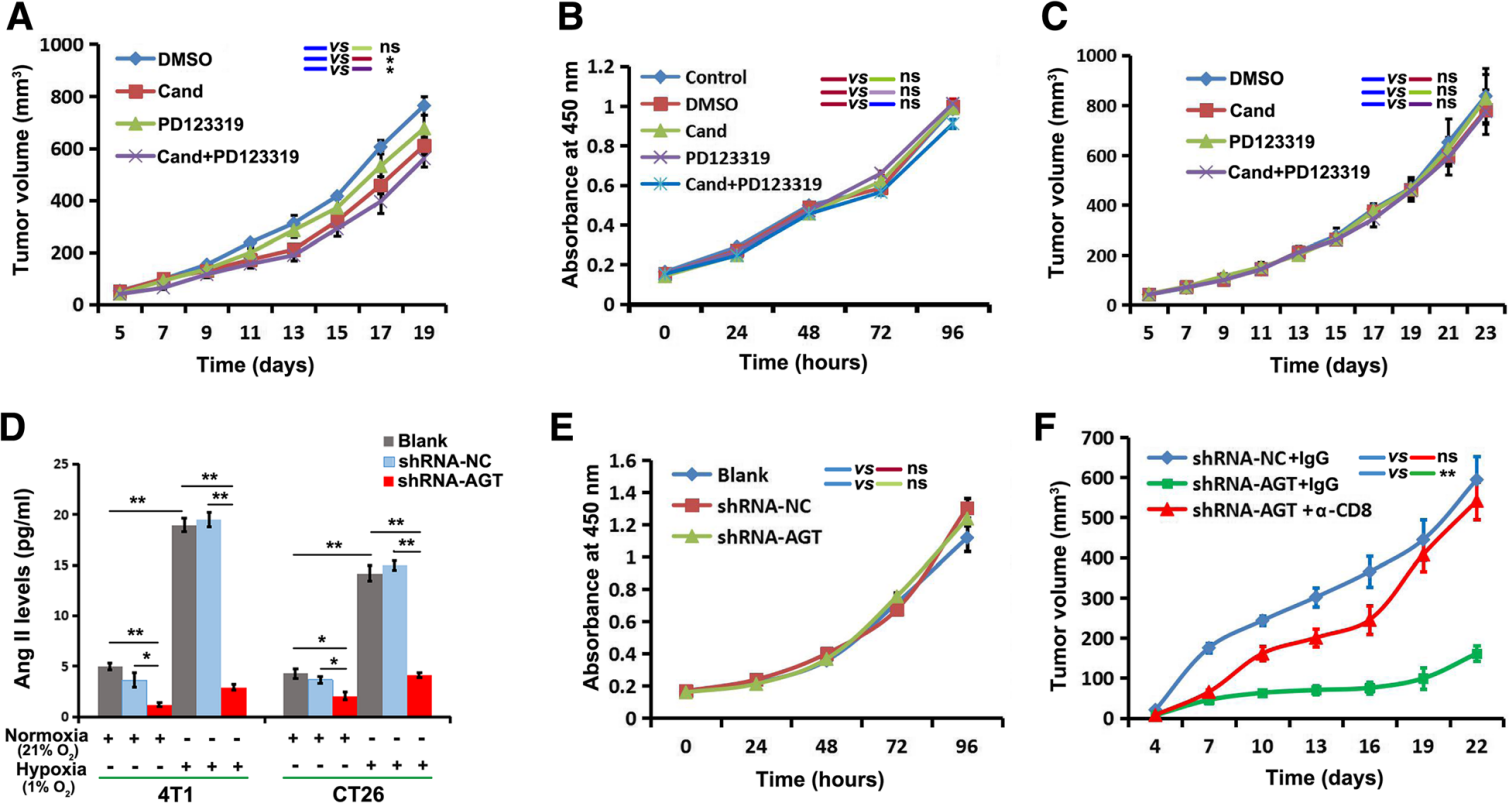
Local AngII in tumor microenvironment is involved in immune escape of tumor cells. **a** The influence of AngII-receptor blockers on 4T1 tumor growth in syngeneic BALB/c mice; candesartan (cand, 5mg/kg, i.p.; starting at day 5 after cell injection, daily ) for AT1R and PD123319 (5mg/kg, i.p.; starting from day 5 after cell injection, every other day) for AT2R, *n*=5. **b** The influence of AngII-receptor blockers on 4T1 cells proliferation in vitro as detected by MTT assay; candesartan (10μm/well) and PD123319 (10μm/well). **c** The influence of Ang II-receptor blockers on 4T1 tumor growth in T-cell immunodeficient BALB/c nude mice, *n*=5. **d** The expression of AGT, a precursor of AngII, was silenced in 4T1 cells and CT26 cells by shRNA. Hypoxia remarkably induced generation of Ang II in 4T1 and CT26 cells as detected by ELISA. AGT-silencing greatly decreased Ang II levels, especially under hypoxic condition. Data are presented as mean±SEM, *n*=3. **e** AGT silencing did not influence cell-proliferation of 4T1 cells in vitro as detected by MTT assay. **f** AGT silencing obviously inhibited tumor growth of 4T1cells in BALB/c mice comparing to negative control, and the depletion of CD8^+^ T cells in mice by in vivo administration of monoclonal antibody against CD8 greatly attenuated this effect (*n*=6). Ns, no significance; *, *P* < 0.05; **, *P* < 0.01

We first established syngeneic tumor models with 4T1 breast cancer cells in immune-competent BALB/c mice. To test the effect of AngII signaling on the 4T1 tumors, BALB/c mice bearing 4T1 tumors of moderate sizes were repeatedly treated with the AngII-receptor blockers candesartan for AT1R and PD123319 for AT2R. Although 4T1 tumor growth was slightly retarded by PD123319, significant inhibition of tumor growth was only observed when mice were treated by candesartan alone or a combination of them (Fig. 1a). To determine whether the anti-tumor growth effect of AngII signaling blockage was caused by directly inhibiting the proliferation of the 4T1tumor cells, the effect of AngII signaling blockage on 4T1 cell proliferation was evaluated in vitro by a MTT assay. We observed no difference in cell proliferative ability in vitro between the cells treated with candesartan, PD123319, combination of both, and DMSO (Fig. 1b). Furthermore, we performed the same in vivo experiment using BALB/c nude mice that were T-cell immunodeficient. Neither candesartan nor PD123319 could inhibit tumor growth in these T-cell immunodeficient mice (Fig. 1c). These results indicate that AngII signaling may be involved in the immune escape of 4T1 tumor cells in BALB/c mice.

Next, to demonstrate the role of local AngII in mediating the immune escape of tumor cells in BALB/c mice, the expression of AGT, a precursor of AngII, was stably silenced in 4T1 breast cells and CT26 colon cancer cells by lentiviral vector-mediated short hairpin RNA (shRNA), and the efficacy of the knockdown was shown in Additional file 1: Figure S1. We examined AngII expression in hypoxic and normoxic conditions in 4T1 and CT26 cells with or without expression of AGT shRNA. Consistent with our previous report [16], hypoxia remarkably induced AngII generation in 4T1 and CT26 cells as detected by ELISA (Fig. 1d). The inhibition of AGT expression significantly decreased AngII levels in the supernatant of these cultured tumor cells under both normoxic and hypoxic condition (Fig. 1d). Similar to ATR blockage, AGT silencing did not influence cell proliferation in vitro as detected by MTT assay (Fig. 1e) and tumor growth in our immunodeficient mouse model (NOD/SCID mice) (Additional file 1: Figure S2A and B). However, AGT silencing in these cells clearly inhibited the tumor growth in 4T1 breast cancer and CT26 colon cancer in BALB/c mice with normal immune systems (Fig. 1f and Additional file 1: Figure S2C). Furthermore, we conducted a CD8 T cell depletion experiment by in vivo administration of a monoclonal antibody against CD8 and found that the differential tumor growth in BALB/c mice disappeared for both 4T1 and CT26 cells (Fig. 1f and Additional file 1: Figure S2C). These results indicate that AngII may contribute to immune escape of 4T1 or CT26 cells in BALB/c mice through suppressing a CD8 T-cell mediated anti-tumor immune response.

### AngII signaling blockage sensitizes tumors to checkpoint immunotherapy

**Fig. 2:**
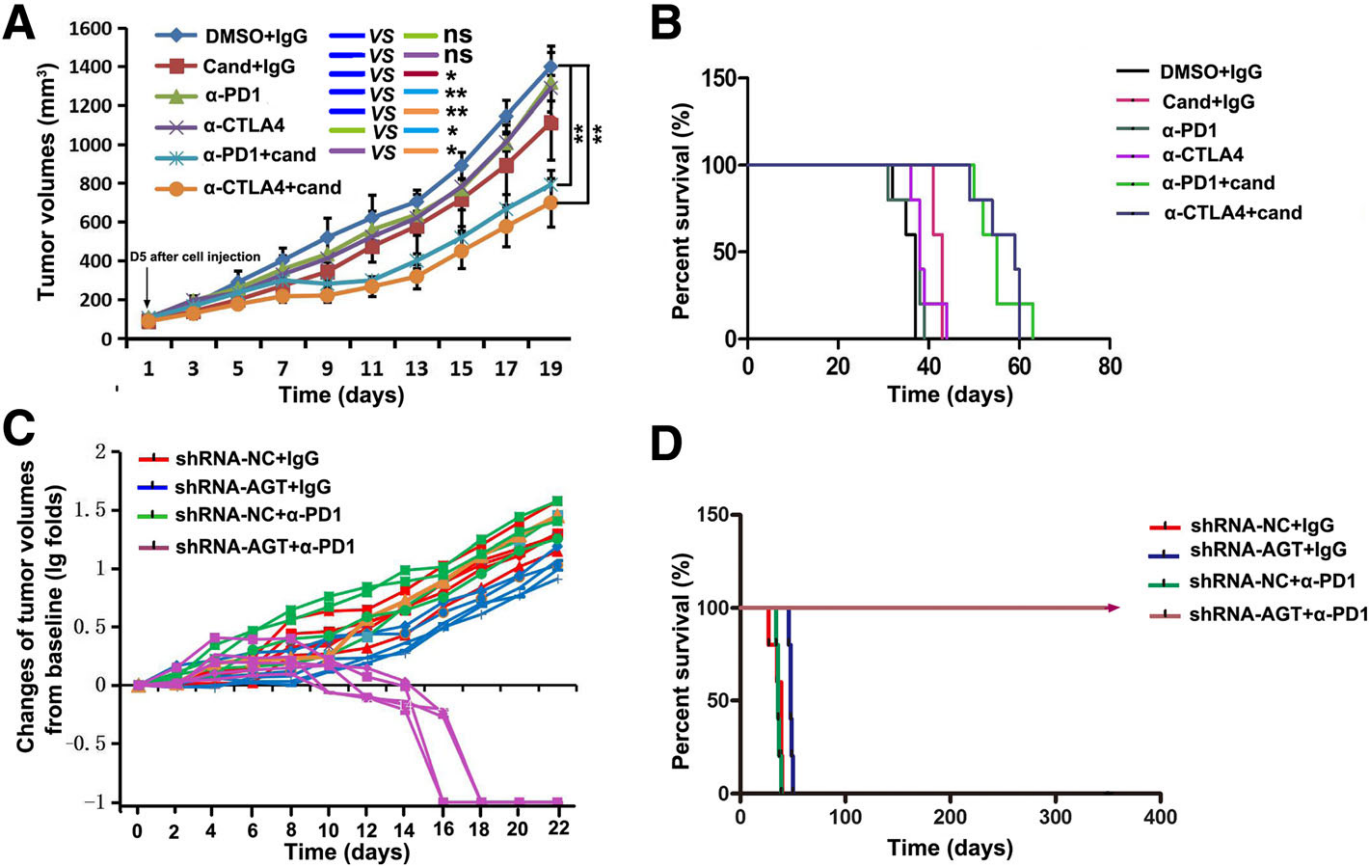
AngII signaling blockage sensitizes tumors to checkpoint immunotherapy in mice tumor models. **a** No significant inhibition of tumor growth when treated with anti-PD-1(α-PD1) or anti-CTLA-4 (α-CTLA4) alone; however, candesartan remarkably improved the effect of immune checkpoint blockers on 4T1 tumors (*n*=5 ); ATR blockers and checkpoint inhibitors were administered starting from day 5 after cell injection. **b** Checkpoint immunotherapy combined with candesartan significantly prolonged the survival of 4T1 tumor-bearing mice ( *n*=5 ). **c** AGT gene-silencing (shRNA-AGT) in 4T1 cells rendered tumors completely responsive to PD1 checkpoint immunotherapy; negative control: shRNA-NC. **d** AGT gene-silencing in 4T1 tumors combined with PD1 immunotherapy made all of mice (*n*=5) achieve long-term survival without any recurrence during almost one-year observation. Ns, no significance; *, *P* < 0.05; **, *P* < 0.01

Because it has been well-documented that 4T1 tumors are highly resistant to PD1 and CTLA-4 checkpoint immunotherapy [26, 30], we sought to determine whether an AngII signaling blockage could enhance the response of 4T1 tumors to checkpoint immunotherapy. BALB/c mice with 4T1 tumors were repeatedly treated by intraperitoneal injection (i.p.) with anti-PD-1 or anti-CTLA-4 antibodies as single agents or in combination with candesartan. We did not observe any significant inhibition of tumor growth when they were treated with anti-PD-1 or anti-CTLA-4 alone (Fig. 2a ). Interestingly, candesartan could remarkably improve the effect of these immune checkpoint blockers on 4T1 tumors (Fig. 2a ) and prolonged the survival of 4T1 tumor-bearing mice (Fig. 2b ). To further verify this effect of AngII signaling blockage on checkpoint immunotherapy, BALB/c mice bearing AGT-silenced 4T1 tumors were also treated by intraperitoneal injection of anti-PD-1 antibody (Additional file 1: Figure S3). After four administrations of anti-PD-1 antibody, all observable tumors derived from AGT-silenced 4T1 cells completely disappeared (Fig. 2c and Additional file 1: Figure S4A). More importantly, no recurrence was noted in these mice which have achieved long-term survival over one-year of observation (Fig. 2d). Similar results were obtained in the CT26 colon tumor model in syngeneic BALB/c mice (Additional file 1: Figure S4B). These results suggest that Ang II signaling blockage sensitizes tumors to checkpoint immunotherapy in mouse models.

### AngII signaling blockage reverses the immunosuppressive tumor microenvironment

**Fig. 3:**
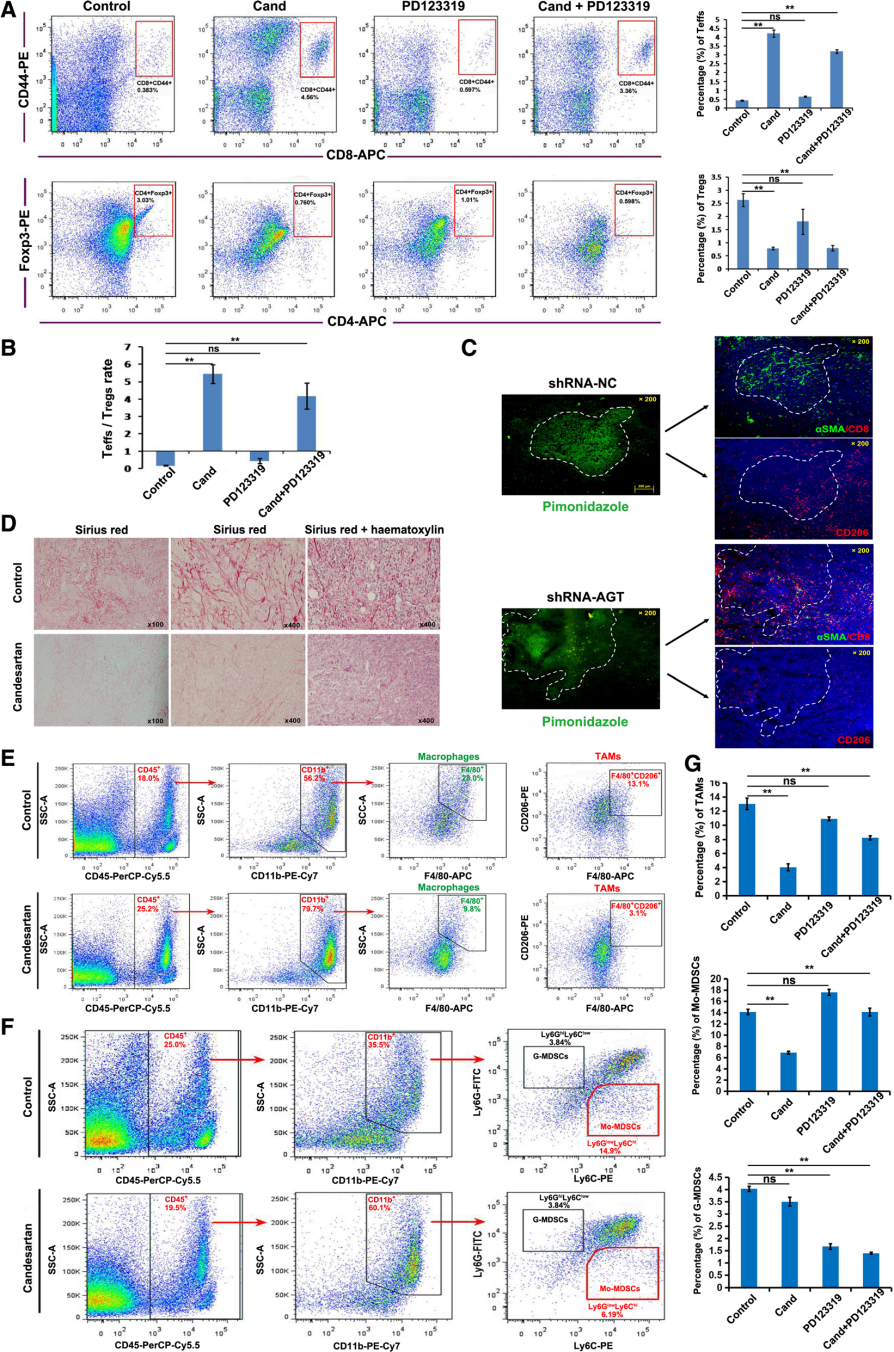
AngII signaling blockage reverses immunosuppressive tumor microenvironment. **a** Representative FACS plot of T_eff_ (CD8^+^CD44^+^) and T_reg_ (CD4^+^Foxp3^+^) in 4T1 breast tumors from BALB/c mice treated with different Ang II-receptor blockers. Bar chart (right) indicated statistic difference (**, *P* < 0.01; ns, no significance). Data are presented as mean±SEM, *n*=3. **b** Bar chart indicated T_eff_/T_reg_ rate (**, *P* < 0.01). Data are presented as mean±SEM, *n*=3. **c** Immunofluorescence analysis showed lots of α-SMA positive fibroblasts and less CD8-positive T cells in hypoxic regions ( pimonidazole-positive ) of 4T1 tumors. AGT gene silence in 4T1 cells obviously inhibited the infiltration of fibroblasts and increased CD8-positive T cells. There were an accumulation of CD206-positive (a marker of mouse M2-phenotype TAMs) cells in pimonidazole-positive hypoxic regions of 4T1 tumors while AGT gene silence remarkably decreased the infiltration of CD206-positive cells in 4T1 tumors, especially in hypoxic regions. **d** Candesartan abated collagen fiber deposition in 4T1 tumors as detected by Sirius Red staining. **e** Representative FACS plot of TAMs (CD45^+^CD11b^+^F4/80^+^CD206^+^) in 4T1 tumors with or without candesartan treatment. **f** Representative FACS plot of Mo-MDSCs (CD45^+^CD11b^+^Ly6G^low^Ly6C^high^) and G-MDSCs (CD45^+^CD11b^+^Ly6G^high^Ly6C^low^) in 4T1 tumors with or without candesartan treatment. **g** Bar chart indicated the percentages of TAMs, Mo-MDSCs and G-MDSCs in 4T1 tumors (**, *P* < 0.01). Data are presented as mean ± SEM, *n*=3

To explore the potential mechanism by which an AngII signaling blockage enhances tumor responses to checkpoint immunotherapy, we first performed multicolor flow cytometry analysis (FACS) to detect the proportions of effector T cells ( T_effs_ ) and regulatory T cells ( T_regs_ ) in 4T1 breast tumors from BALB/c mice treated with Ang II-receptor blockers. We found that candesartan significantly increased the frequencies of CD3^+^ T cells in 4T1 tumors (Additional file 1: Figure S5A). More importantly, candesartan remarkably increased the frequencies of CD8^+^CD44^+^ T_eff_ cells and decreased that of CD4^+^Foxp3^+^ T_reg_ cells in 4T1 tumors (Fig. 3a), leading to an increased T_effs_/T_regs_ ratio (Fig. 3b). Similar results were also observed in tumors treated with a combination of candesartan and PD123319. However, PD123319 alone did not increase the frequencies of T_eff_ cells in tumors, but it non-significantly decreased the frequency of T_reg_ cells compared to the control tumors (Fig. 3a and b). Furthermore, AGT expression suppression in tumor cells had the same impact on tumor infiltrating T cells as the candesartan treatment (Additional file 1: Figure S6). These results indicate that AngII signaling, predominantly Ang II-AT1R-mediated signaling, may be involved in forming a tumor immunosuppressive microenvironment .

Since the pathogenesis of AngII in chronic organ diseases is largely mediated by inducing the infiltration and activation of fibroblasts [17, 18] and macrophages [19, 20], we sought to examine whether AngII mediates infiltration and activation of fibroblasts and macrophages in 4T1 tumors. Immunofluorescence analysis showed many α-smooth muscle actin (α-SMA)-positive fibroblasts and fewer CD8-positive T cells in 4T1 breast tumors, especially in pimonidazole-positive ( a probe for tumor hypoxia ) hypoxic regions (Fig. 3c and Additional file 1: Figure S7 ). However, AGT-silencing remarkably inhibited the infiltration of fibroblasts and increased the number of CD8-positive T cells in 4T1 tumors (Fig. 3c and Additional file 1: Figure S7). We further measured fibrosis of 4T1 tumors and found a high degree of collagen fiber deposition in 4T1 breast tumors (Fig. 3d). Interestingly, candesartan abated collagen fiber deposition in 4T1 tumors as detected by Sirius Red staining (Fig. 3d).

We also found that there was an a decrease in CD206-positive (a marker of mouse M2-phenotype TAMs) cells in pimonidazole-positive hypoxic regions of 4T1 tumors upon ATG silencing (Fig. 3c and Additional file 1: Figure S7). These results indicate that AngII signaling could be involved in infiltration of TAMs in tumors. Next, we used multicolor flow cytometry to analyze the content of TAMs (CD45^+^CD11b^+^F4/80^+^CD206^+^) in 4T1 tumors. We found that candesartan treatment significantly inhibited the accumulation of macrophages (CD45^+^CD11b^+^F4/80^+^) and TAMs in 4T1 tumors compared to tumors from control mice without candesartan treatment (Fig. 3e and g, and Additional file 1: Figure S5B). We also found that the frequency of monocytic myeloid-derived suppressor cells (Mo-MDSCs, CD45^+^CD11b^+^Ly6G^low^Ly6C^high^) remarkably decreased in candesartan-treated tumors compared to those from control mice, and however, the decrease in the proportions of granulocytic myeloid-derived suppressor cells (G-MDSCs) in tumors upon candesartan treatment was non-significant (Fig. 3f and g, and Additional file 1: Figure S5C). Consistently, we observed that in AGT-silenced 4T1 tumors, the number of TAMs obviously decreased compared with control 4T1 tumors (Additional file 1: Figure S8A and C ). Notably, AGT-silenced 4T1 tumors exhibited decreased percentages of both Mo-MDSCs and G-MDSCs, compared to control 4T1 tumors (Additional file 1: Figure S8B and C). These results further support our hypothesis that local AngII contributes to the formation of a tumor immunosuppressive microenvironment and the AngII signaling blockage reverses it into an immune-activating microenvironment.

### Inhibition of AGT expression strongly triggers an immune-activating cytokine profile in hypoxic 4T1 cells

**Fig. 4:**
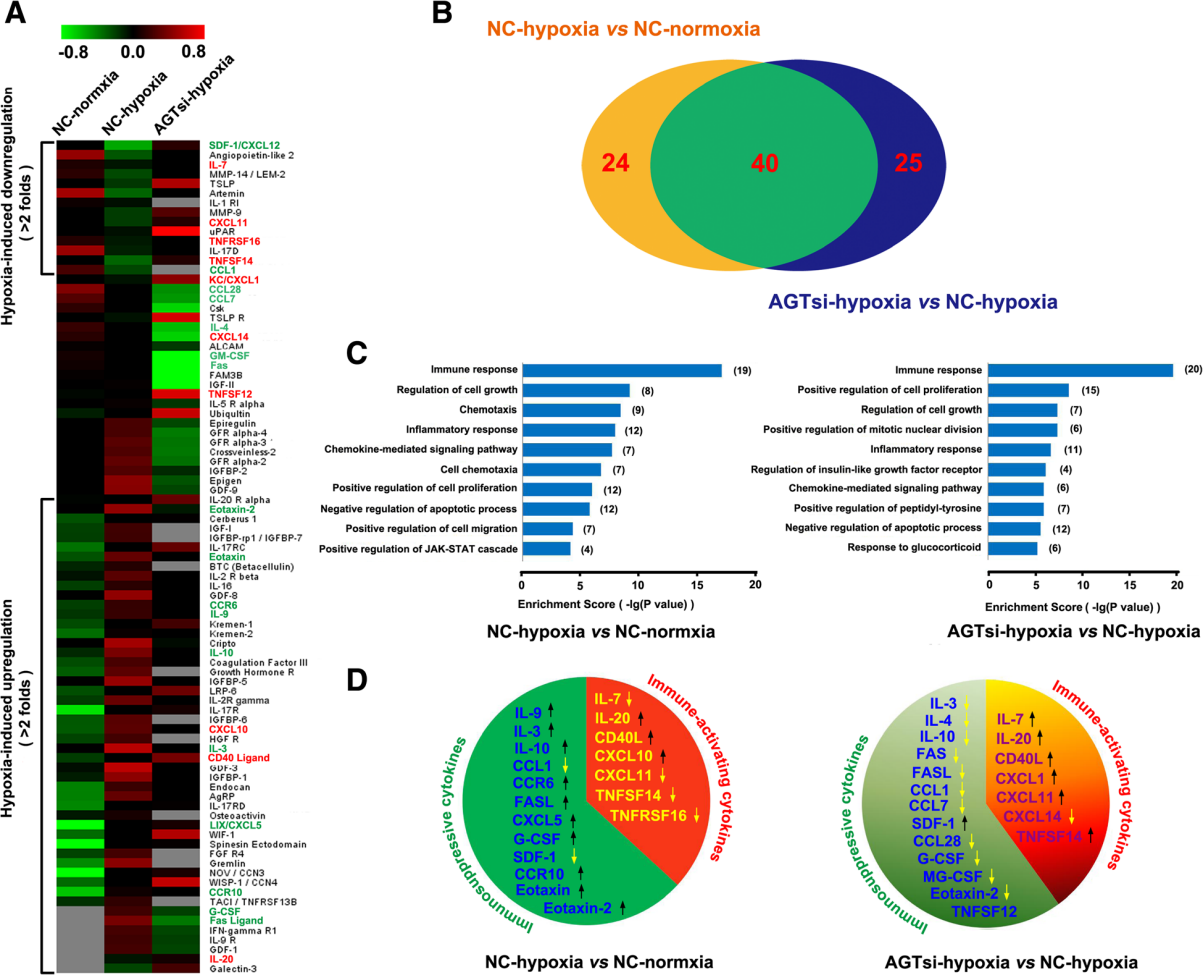
AGT expression silencing triggers an immune-activating cytokine profile in hypoxic 4T1 cells. **a** Cytokine profiles ( containing 308 cytokines ) of AGT-silenced 4T1 cells (AGTsi) or negative control (NC) in normoxia or hypoxia condition were analyzed using RayBio Mouse Cytokine Antibody Array. Heat-map displayed differential cytokine expression (>2 folds). **b** The cytokines (64) impacted by AGT-silencing were largely overlaped (40 cytokines) with those (65) influenced by hypoxia exposure. **c** Functional relationships of the influenced cytokines (>2folds) according to Gene Ontology (GO) analysis. **d** The levels of immune-activating cytokines and immunosuppressive cytokines were altered by hypoxia and AGT silencing

Accumulating evidence has revealed that intratumor hypoxia contributes to tumor immune escape by altering the function of innate and adaptive immune cells or by increasing the intrinsic resistance of tumor cells to cytolytic activity of immune effectors [31–33]. To demonstrate the mechanism of immune activation by AngII signaling blockage, cytokine profiles (containing 308 cytokines) of 4T1 cells in normoxia or hypoxia were analyzed using the RayBio Mouse Cytokine Antibody Array. Hypoxia induced obvious alterations ( more than 2 folds ) of 64/308 cytokines expression, in which 14 cytokines decreased and 50 increased (more than 2-fold changes) (Fig. 4a). Notably, AGT silencing remarkably altered expression of 65 cytokines in 4T1 hypoxic cells compared with control hypoxic cells. In these 65 cytokines, 45 cytokines were obviously decreased (more than 2 folds) and 20 were increased (more than 2 folds) in hypoxic AGT-silenced 4T1 cells (Fig. 4a). Interestingly, the cytokines altered by AGT-silencing were largely overlapped with those influenced by hypoxia exposure (Fig. 4b). To verify the reliability of the cytokine array data, we further used ELISA analysis to detected the levels of 6 defferential cytokines (IL-10, GM-CSF, G-CSF, Eotaxin-2, CXCL11, and TNFSF14) in the culture supernatant of 4T1 cells under different O_2_ conditions, and results consistent with the cytokine array were obtained for all of 6 cytokines (Additional file 1: Figure S9A).

To investigate functional relationships of the altered cytokines, Gene Ontology (GO) analysis was used to organize the cytokine array data into their functional relationships based on biologic processes and pathways. Hypoxia induced significantly higher frequencies of cytokines which were associated with 39 biological processes (*p*<0.01) (Additional file 1: Figure S9B) and 17 signaling pathways (*p*<0.05) (Additional file 1: Figure S9D). On the other hand, the cytokines altered by AGT-silencing in hypoxia were associated with 58 biological processes (*p*<0.01) (Additional file 1: Figure S9C) and 22 signaling pathways (*p*<0.05) (Additional file 1: Figure S9D). Notably, hypoxia particularly influenced immune response (*p=*9.54E-18) (Fig. 4c), leading to a decreased expression of immune-activating cytokines and an increased expression of immunosuppressive cytokines (Fig. 4d). It is worth noting that AGT silencing also prominently impacted the immune response with differential expression (p=2.04E-20) of 20 cytokines (Fig. 4c). Seven of these 20 cytokines, namely, IL-7, IL-20, CD40 ligand, CXCL1, CXCL11, TNFSF12 and TNFSF14, which have been previously reported to be immune-activating cytokines (Fig. 4d and Table 1), had a remarkably increased expression (changes>2fold). In contrast, 11 of these 20 cytokines, namely, IL-3, IL-10, IL-4, Fas, Fas ligand (FASL), CCL1, CCL7, CCL28, G-CSF, GM-CSF and Eotaxin-2, which have been previously reported to be immunosuppressive cytokines (Fig. 4d and Table 1), had a significantly decreased expression (changes>2fold). These results suggest that hypoxia contributes to the formation of an immunosuppressive microenvironment, and AGT silencing, however, reverses this immunosuppressive status and induces an immune-activating anti-tumor microenvironment.

### AGT silencing combined with PD-1 blockage generates an abscopal effect to resistant tumors

**Fig. 5:**
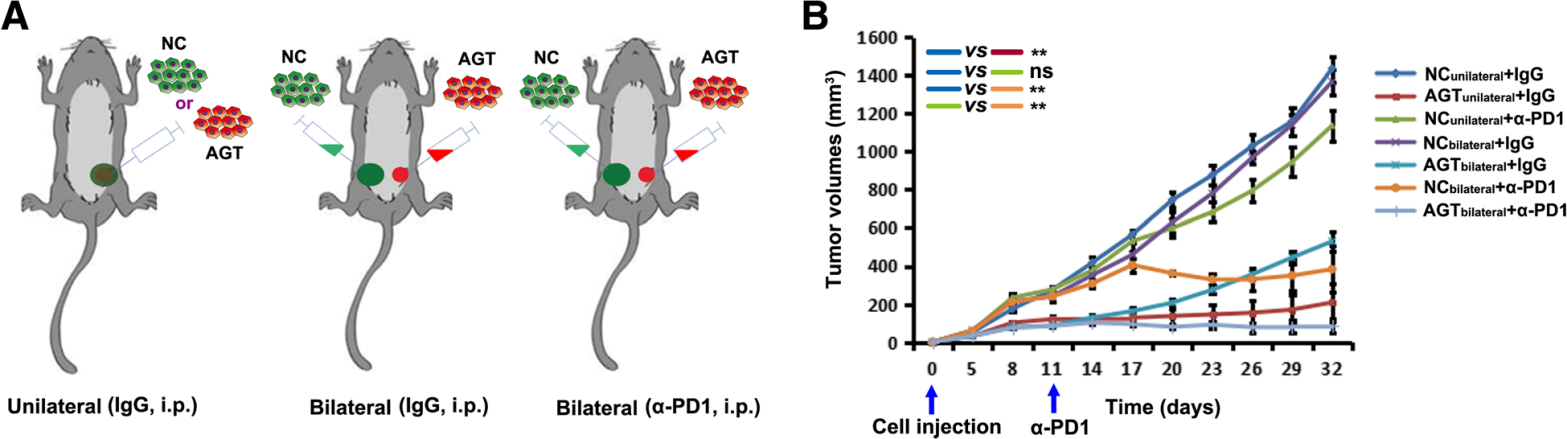
An abscopal effect on resistant tumors is induced by AGT silencing in local tumor combined with PD-1 blockage. **a** AGT-silenced 4T1 cells (AGT) and negative control (NC) were injected into single (unilateral) or two sides (bilateral) respectively of mammary fat pads in BALB/c mice. **b** 4T1 tumor-bearing mice were treated as indicated. The volume of each tumor was measured from each mouse (*n*=6) . Ns, no significance; **, *P* < 0.01

To investigate whether the improved immune microenvironment in a tumor would contribute to the response of other tumors in the same mouse, 4T1 cells with AGT silencing and a negative control (NC) were respectively injected into two sides (bilateral) of mammary fat pads in BALB/c mice (Fig. 5a). We found that AGT-silenced tumors did not influence the growth of contralateral NC tumors in bilaterally-injected mice (Fig. 5b). However, when anti-PD-1 antibody was administrated to bilaterally-injected mice by i.p. injection, NC-tumor growth in the same mice was remarkably inhibited, with NC tumors in three of six mice completely disappearing (Fig. 5b). These results suggests that AGT silencing in tumor combined with system PD-1 blockage results in an abscopal effect on other tumor focuses that were originally resistant to PD-1 blockage in mice.

## Discussion

Currently, immunotherapy by checkpoint inhibition holds great promise for improving cancer patient outcomes, as it has done for patients with melanoma [34] or lung cancer [35]. Unfortunately, checkpoint inhibitors have only achieved limited clinical benefit to date for most malignant tumors, likely due to multiple immune escape mechanisms mediating cancer resistance to immunotherapy [6].

Fibrosis and infiltration of multiple immune suppressor cells in tumors have been suggested as important mechanisms by which a tumor immunosuppressive microenvironment is established in many kinds of human tumors [6], especially in breast cancer [36, 37] and pancreatic cancer [38, 39], which are commonly resistant to current anti-checkpoint immunotherapy. Recent studies have demonstrated that hypoxia plays a critical role in the formation of a tumor immunosuppressive microenvironment by multiple mechanisms [40], but predominantly by increasing the infiltration of CAFs and TAMs in hypoxic tumor areas [40–42]. Our previous report showed that local AngII existed in a tumor hypoxic microenvironments where AngII was produced by hypoxic tumor cells in a hypoxia-lactate-chymase-dependent mechanism, independent from the AngII in the blood circulation system [16]. Herein, using a mouse 4T1 breast cancer model and CT26 colon cancer model, we revealed that AngII generated by hypoxic tumor cells had an important role in forming a tumor immunosuppressive microenvironment.

Other previous studies have shown that AngII could induce the infiltration and activation of fibroblasts and some inflammatory cells, and thus resulted in a variety of chronic organ diseases [17–20]. We hypothesize that the AngII present in tumors may function similarly, and it therefore mediates the recruitment and activation of fibroblasts and inflammatory cells in the tumor microenvironment to support immunosuppression. Herein we found that AngII induced the infiltration of fibroblasts and macrophages into a tumor microenvironment, and the abrogation of AngII generation or AngII signal blockage decreased the infiltration of fibroblasts and macrophages and contributed to CD8^+^ T cell infiltration into tumor regions. These results suggest that AngII seems to play a similar function in tumors as in chronic organ diseases, resulting in an intratumor immunosuppressive microenvironment. A high-content cytokine array ( including 308 cytokines ) analysis showed hypoxia induced an immunosuppressive cytokine profile; however, AGT silencing re-established an immune-activating cytokine profile for hypoxic 4T1 breast cancer cells. Therefore, AngII may confer to these tumor cells the ability to create an immunosuppressive microenvironment so as to escape immune elimination.

AngII signal blockage or AGT expression silencing in tumor cells destroys the tumor immunosuppressive microenvironment and improves the tumor response to immune checkpoint inhibitors. Interestingly, silencing AGT expression in 4T1 cells achieved a long-term tumor remission in all of the mice treated with PD-1 checkpoint inhibitor and induced a relapse-free survival. More importantly, 4T1 mouse tumors with AGT expression silencing made a previously unresponsive control tumor, growing on other site of the mouse, achieve a dramatic response to checkpoint antagonists. These results suggest that the alteration of the immune microenvironment in a local tumor through AGT silencing can trigger a systemic anti-tumor immune response although anti-PD-1 is required to eliminate the tumor cells. A possible mechanism for this is that the destruction of the local immunosuppressive microenvironment contributes to tumor antigen presentation and consequently activates multiple antigen-specific effector T cells which are capable of specifically attacking tumor cells at any sites in the body when combined with anti-PD-1 therapy. AGT silencing in hypoxic 4T1 cells triggered an immune-activating cytokine profile, including some cytokines involved in attracting, activating, and stimulating proliferation of dendritic cells and T cells [43–49], which contributes to tumor antigen presentation and generation of multiple tumor-antigen-specific effector T cells. Therefore, the altered immune microenvironment in a local tumor by AGT expression-silencing may elicit an in situ tumor vaccination and generate productive tumor-specific T cells to achieve a systemic in vivo anti-tumor effect. Given the role of AngII signaling in inhibiting HIF-1α protein accumulation in hypoxia tumors [16], another possible mechanism of the benefit from an AngII signaling blockade (both genetic and pharmacological) is that an AngII signaling blockade sensitizes these tumors to effective T cell-mediated killing because hypoxia-induced HIF-1alpha has been shown to prevent CTL-mediated killing [50, 51].

A recently published article by Nakai showed that candesartan failed to improve survival in those patients receiving gemcitabline-based combination chemotherapy [52]. However, a subgroup analysis showed that patients receiving gemcitabine monotherapy could benefit from candesartan [15]. A possible explanation for the negative results by Nakai is that excessive combination chemotherapy profoundly impairs patients’ immune system, due to drug-cytotoxicity acting on normal immune cells, which could overwhelm any benefit from candesartan.

## Conclusions

In the present study, we demonstrated that local AngII contributed to the formation of a tumor immunosuppressive microenvironment that mediated tumor resistance to current checkpoint immunotherapy. AngII signal blockage or abrogating AngII generation in tumor cells remarkably enhanced the effect of anti-checkpoint immunotherapy in BALB/c mice with a normal immune system. A combination of AngII signal blocker with a checkpoint antagonist could be a promising strategy to improve tumor responses to current anti-checkpoint immunotherapy.

## Additional file

Additional file 1: **Figure S1.** The efficacy of knockdown was detected through qRT-PCR and western blotting. The efficacy of knockdown in 4T1 cells (A) and CT26 cells (B). **Figure S2.** Role of AGT gene-silencing in growth of 4T1 and CT26 cells. AGT-silencing didn't significantly inhibit tumor growth of 4T1 (A) and CT26 cells (B) in NOD/SCID mice. (C) AGT silencing inhibited CT26 growth in BALB/c mice and the depletion of CD8^+^ T cells reversed this role. **Figure S3.** Strategy of combined AGT gene-silencing and PD1 blockade. **Figure S4.** AngII signaling blockage sensitizes tumors to checkpoint immunotherapy. AGT gene-silencing in 4T1 (A) and CT26 cells (B) rendered tumors more sensitive to anti-PD1 immunotherapy. **Figure S5.** Percentages of CD3^+^, CD45^+^, and CD11b^+^ cells in 4T1 tumors from BALB/c mice treated with different Ang II-receptor blockers. (A), (B), and (C) correspond to **Figure 3A**, **3E**, and **3F**. **Figure S6.** Representative FACS plot of T_effs_ (CD8^+^CD44^+^) and T_regs_ (CD4^+^Foxp3^+^) in AGT-silenced and control 4T1 tumors from BALB/c mice. Bar chart (right) indicated statistic difference (*n* = 3). **Figure S7.** α-SMA, CD8 or CD206 positive cells in hypoxic regions of 4T1 tumors. Positive cells were counted in 4 random 400× microscope visions in hypoxic regions of AGT-silenced or control tumors which were from 3 independent mice, (*n* = 12). **Figure S8.** The content of TAMs, Mo-MDSCs and G-MDSCs in shRNA-AGT 4T1 tumors. (A-B) Representative FACS plot. (C) Percentages of these populations (*n* = 3). **Figure S9.** AGT-silencing triggers an immune-activating cytokine profile in hypoxic 4T1 cells. The levels of 6 cytokinesby ELISA analysis (A). Gene Ontology analysis showed hypoxia induced significantly higher frequencies of cytokines which were associated with 39 biological processes (B, *p* < 0.01) and 17 signaling pathways (D, left, *p* < 0.05). The cytokines influenced by AGT-silencing in hypoxia condition were associated with 58 biological processes (C, *p* < 0.01) and 22 signaling pathways (D, right, *p* < 0.05). **Table S1.** Antibodies for Immunofluorescence. (DOCX 48272 kb)

## Abbreviations

ACE: Angiotensin-converting enzyme
AGT: Angiotensinogen
AngII: Angiotensin II
AT1R: AngII type 1 receptor
AT2R: Ang II type 2 receptor
CAFs: Cancer-associated fibroblasts
CTLA-4: Cytotoxic-T-lymphocyte-associated protein 4
G-MDSCs: Granulocytic myeloid-derived suppressor cells
GO: Gene Ontology
Mo-MDSCs: monocytic myeloid-derived suppressor cells
NC: Negative control
PD-1: Programmed cell death protein 1
RAS: Renin-angiotensin system
shRNA: Short hairpin RNA
TAMs: Tumor-associated macrophages
T_effs_: Effective T cells
T_regs_: Regulatory T cells
α-SMA: α-smooth muscle actin
