# Supplementary material for: Local angiotensin II contributes to tumor resistance to checkpoint immunotherapy
Source: J Immunother Cancer. 2018 Sep 12;6:88. doi: 10.1186/s40425-018-0401-3 (PMC6134794; doi:10.1186/s40425-018-0401-3)
Supplement: Supplementary file 1 — Figure S1. The efficacy of knockdown was detected through qRT-PCR and western blotting. The efficacy of knockdown in 4T1 cells (A) and CT26 cells (B). Figure S2. Role of AGT gene-silencing in growth of 4T1 and CT26 cells. AGT-silencing didn't significantly inhibit tumor growth of 4T1 (A) and CT26 cells (B) in NOD/SCID mice. (C) AGT silencing inhibited CT26 growth in BALB/c mice and the depletion of CD8+ T cells reversed this role. Figure S3. Strategy of combined AGT gene-silencing and PD1 blockade. Figure S4. AngII signaling blockage sensitizes tumors to checkpoint immunotherapy. AGT gene-silencing in 4T1 (A) and CT26 cells (B) rendered tumors more sensitive to anti-PD1 immunotherapy. Figure S5. Percentages of CD3+, CD45+, and CD11b+ cells in 4T1 tumors from BALB/c mice treated with different Ang II-receptor blockers. (A), (B), and (C) correspond to Figure 3A, 3E, and 3F. Figure S6. Representative FACS plot of Teffs (CD8+CD44+) and Tregs (CD4+Foxp3+) in AGT-silenced and control 4T1 tumors from BALB/c mice. Bar chart (right) indicated statistic difference (n = 3). Figure S7. α-SMA, CD8 or CD206 positive cells in hypoxic regions of 4T1 tumors. Positive cells were counted in 4 random 400× microscope visions in hypoxic regions of AGT-silenced or control tumors which were from 3 independent mice, (n = 12). Figure S8. The content of TAMs, Mo-MDSCs and G-MDSCs in shRNA-AGT 4T1 tumors. (A-B) Representative FACS plot. (C) Percentages of these populations (n = 3). Figure S9. AGT-silencing triggers an immune-activating cytokine profile in hypoxic 4T1 cells. The levels of 6 cytokinesby ELISA analysis (A). Gene Ontology analysis showed hypoxia induced significantly higher frequencies of cytokines which were associated with 39 biological processes (B, p < 0.01) and 17 signaling pathways (D, left, p < 0.05). The cytokines influenced by AGT-silencing in hypoxia condition were associated with 58 biological processes (C, p < 0.01) and 22 signaling pathways (D, right, p < 0.05). Table [file 40425_2018_401_MOESM1_ESM.docx]

**Additional file 1**

**Local angiotensin II contributes to tumor resistance to checkpoint immunotherapy**

Guozhu Xie, Tan Cheng, Jie Lin, Lanfang Zhang, Jieling Zheng, Ying Liu, Guobo Xie, Baiyao Wang, Yawei Yuan.

**Supplementary Figures**

**
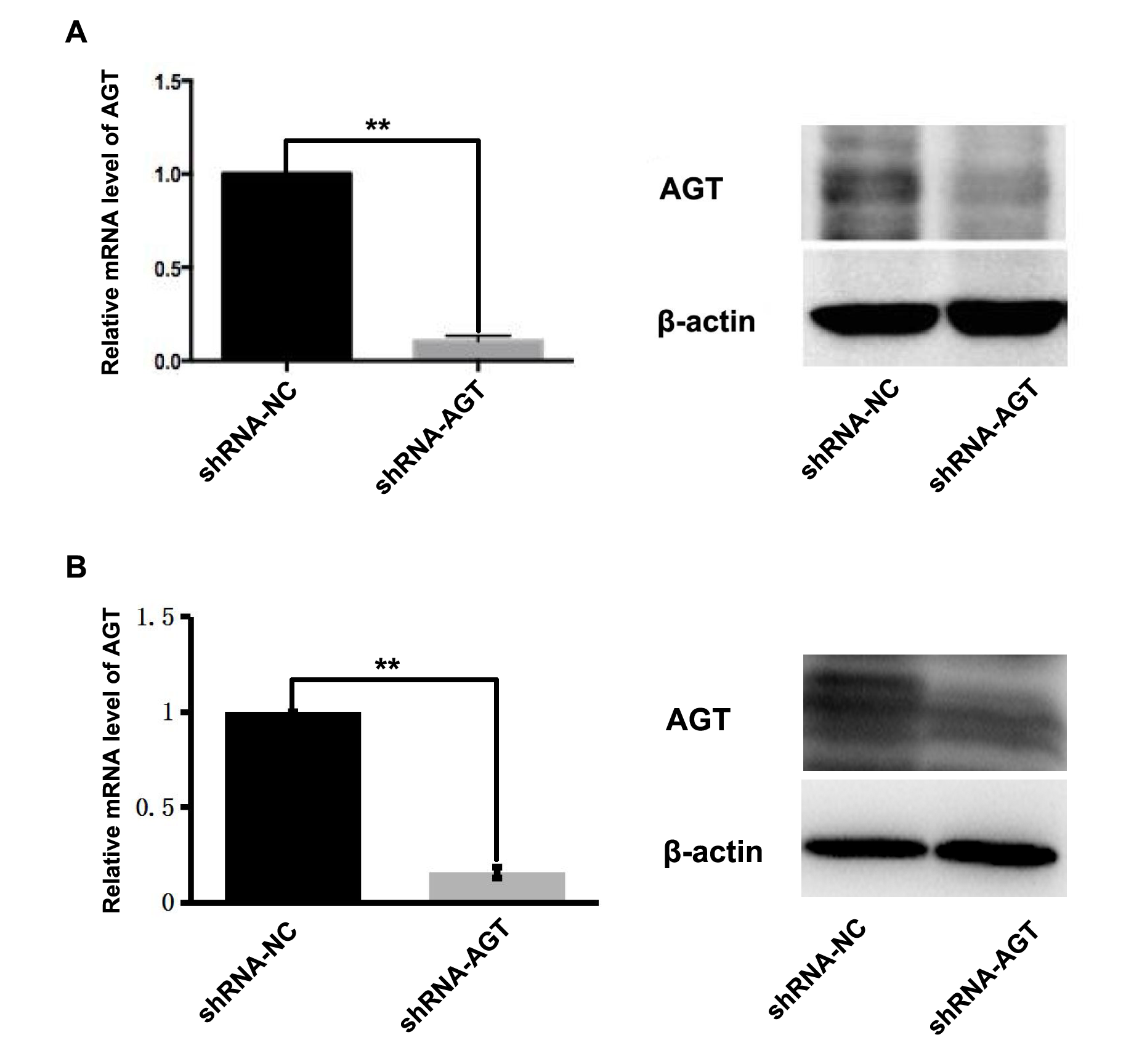
**

**Figure S1** **The efficacy of knockdown by shRNA was detected through qRT-PCR and western blotting. (A) The efficacy of knockdown by shRNA was evaluated in 4T1 cells. (B) The efficacy of knockdown by shRNA was evaluated in CT26 cells.** **, P < 0.01.


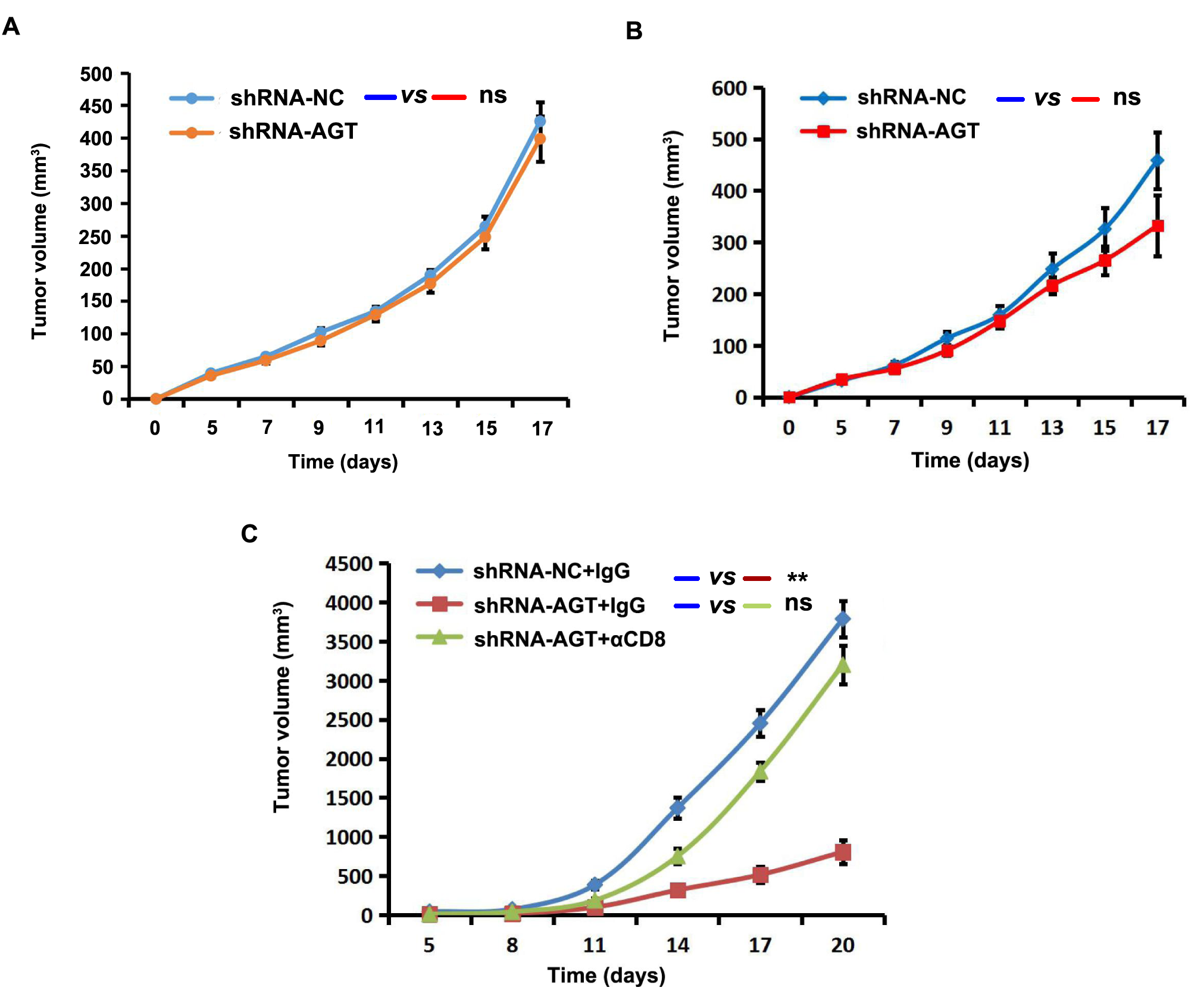


**Figure S2** **Role of AGT gene silence in growth of 4T1 breast cancer and CT26 colon cancer cells.** (A) AGT-silencing did not lead to significant inhibition of tumor growth of 4T1 cells in immunodeficiency mice model (NOD/SCID mice). (B) AGT-silencing did not lead to significant inhibition of tumor growth of CT26 cells in immunodeficiency mice model (NOD/SCID mice). (C) AGT silencing inhibited tumor growth of CT26 colon cancer in BALB/c mice with normal immune system; the depletion of CD8^+^ T cells reversed this inhibiting role of AGT silence. Ns, no significance; **, P < 0.01.


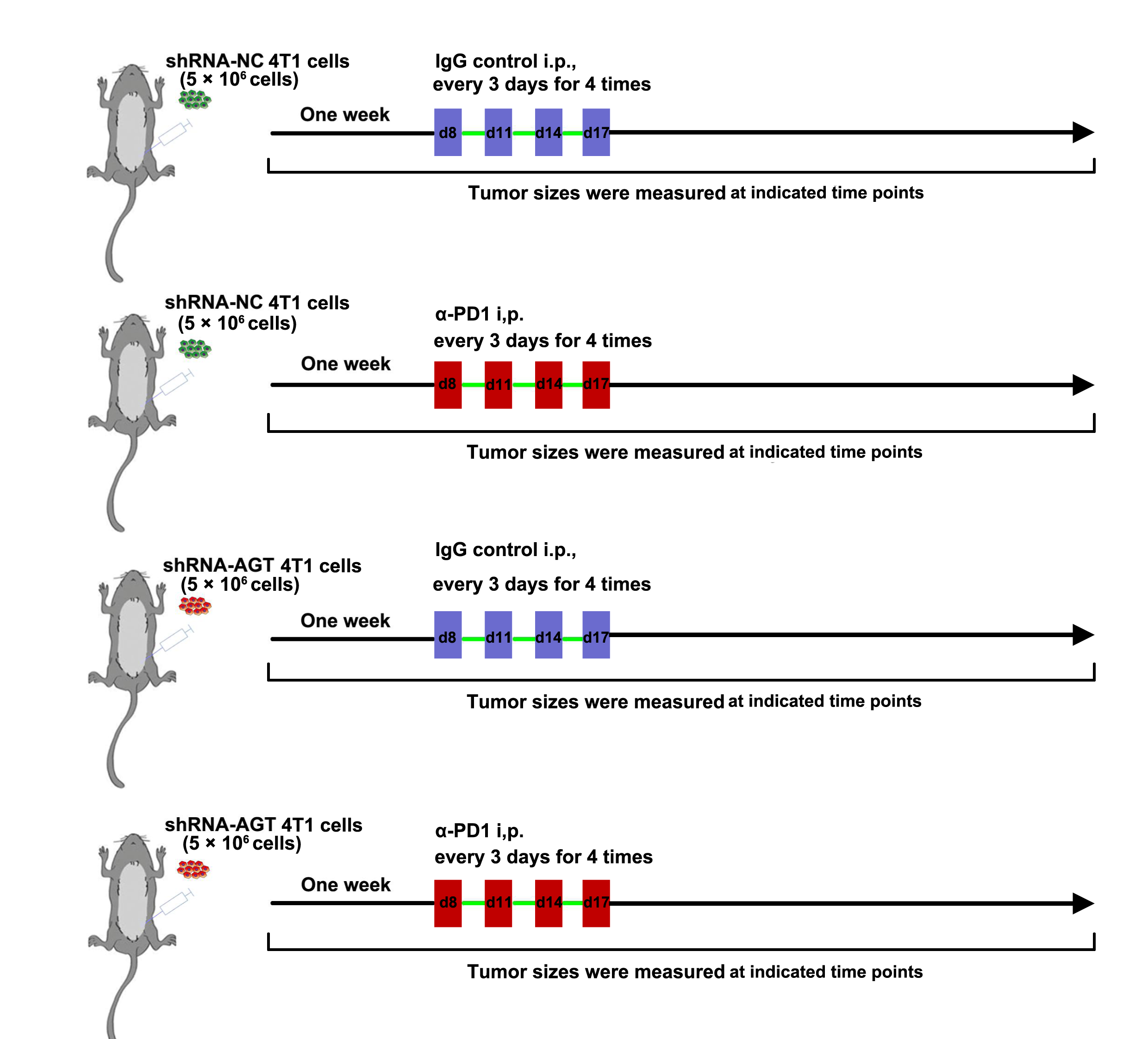


**Figure S3** **Strategy of combined AGT gene-silencing and PD1 blockade.**

**A**


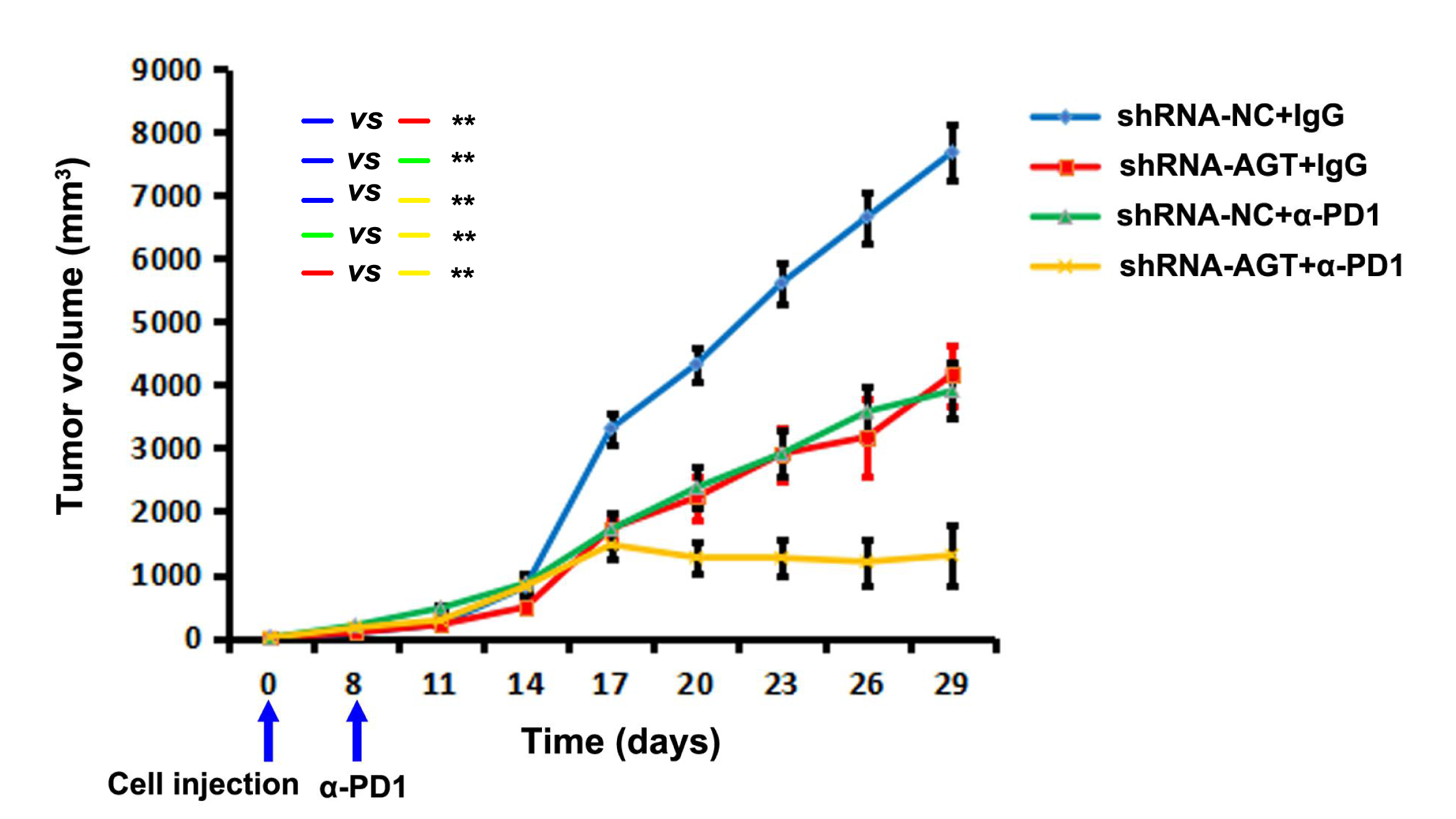

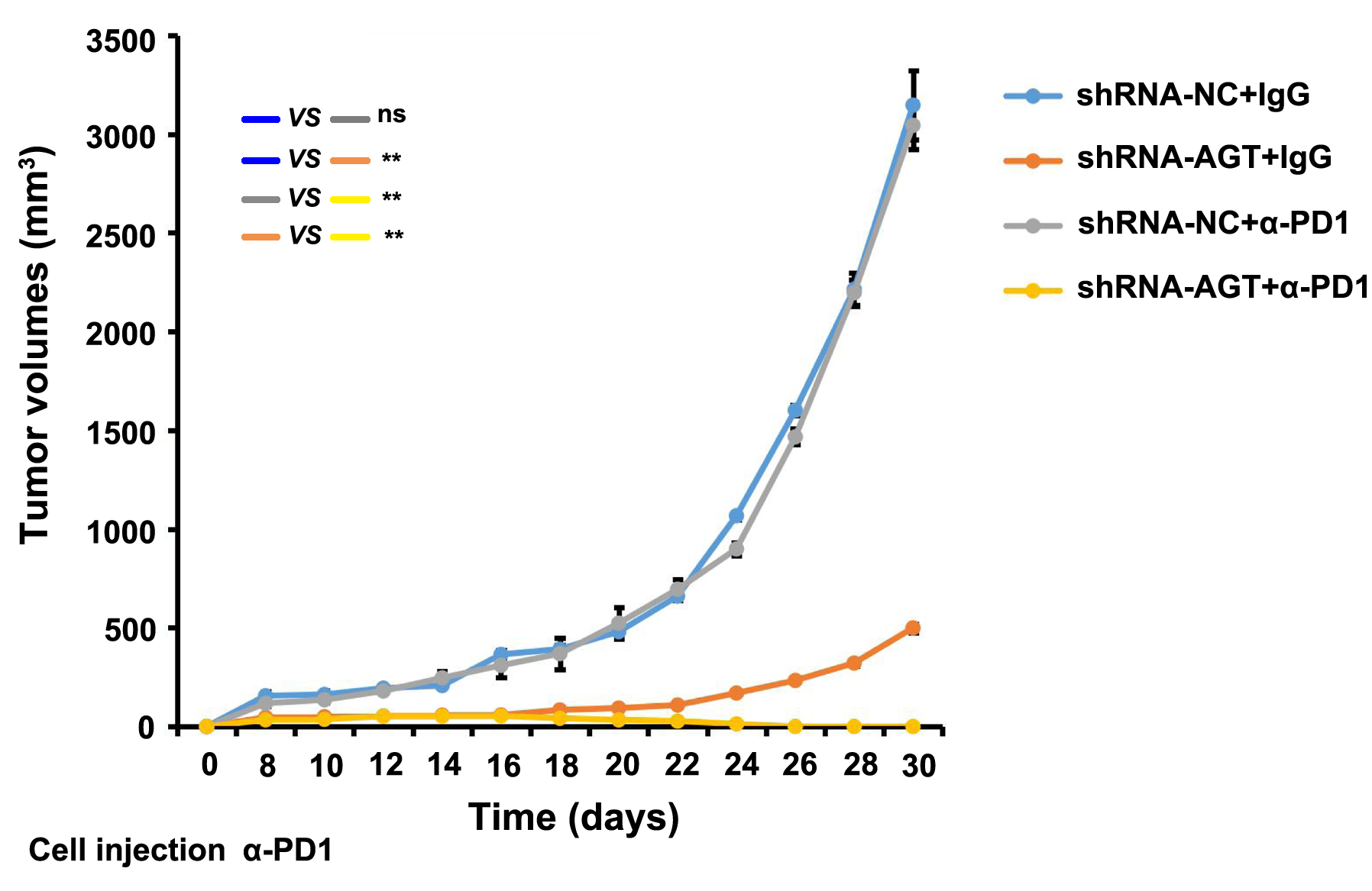


**B**

**Figure S4**  **AngII signaling blockage sensitizes tumors to checkpoint immunotherapy in mice tumor models.** AGT gene-silencing (shRNA-AGT) in 4T1 breast cancer cells (A) and CT26 colon cancer cells (B) rendered tumors more sensitive to PD1 checkpoint immunotherapy; negative control: shRNA-NC; ns, no significance; **, P < 0.01.


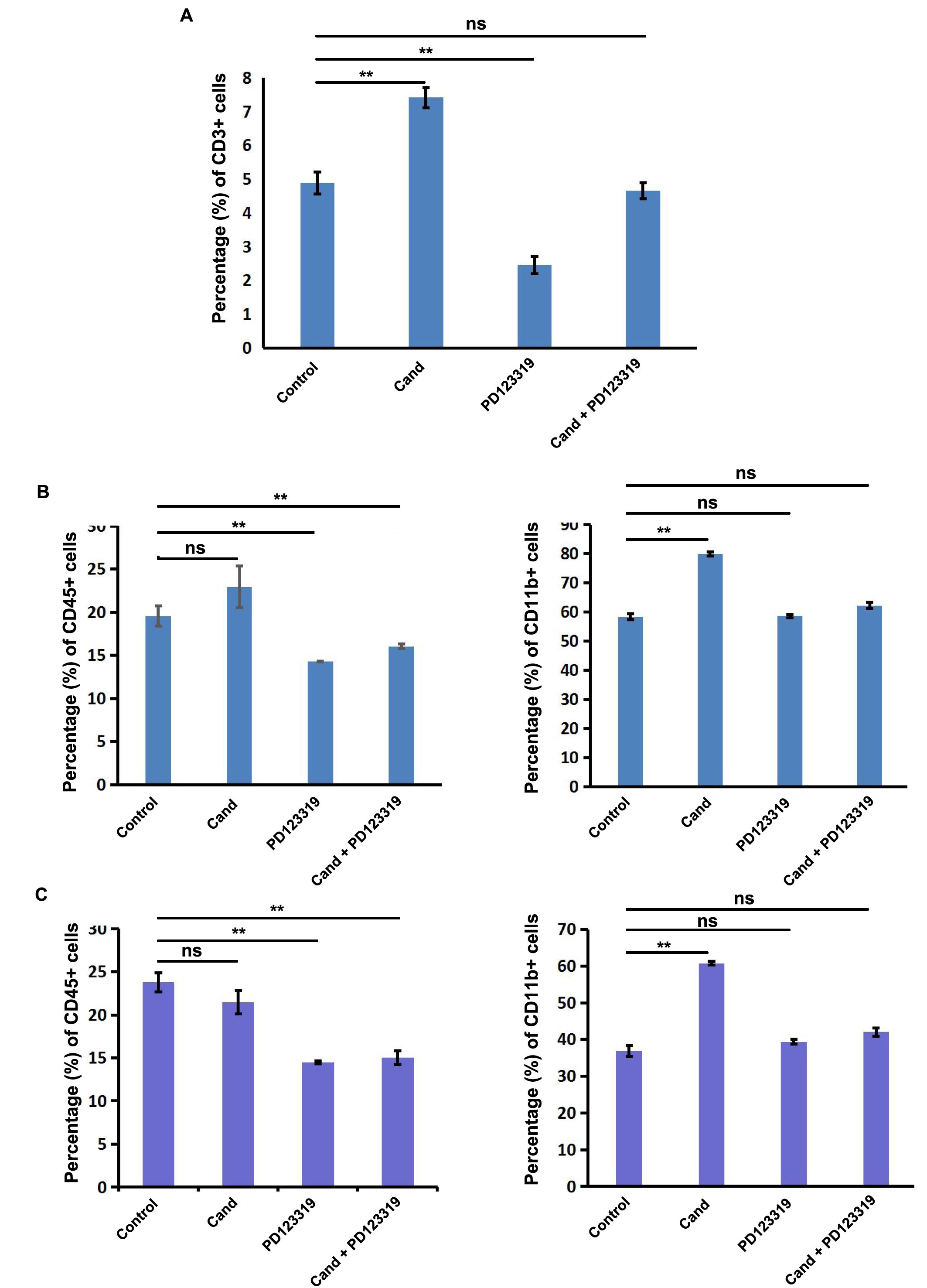


**Figure S5 Percentages of CD3^+^, CD45^+^, and CD11b^+^ cells in 4T1 breast tumors from BALB/c mice treated with different Ang II-receptor blockers.** (A) Percentages of CD3^+^ cells in 4T1 breast tumors from BALB/c mice treated with different Ang II-receptor blockers, corresponding to Figure 3A. (B) Percentages of CD45^+^ and CD11b^+^ cells in 4T1 breast tumors from BALB/c mice treated with different Ang II-receptor blockers, corresponding to Figure 3E. (C) Percentages of CD45^+^ and CD11b^+^ cells in 4T1 breast tumors from BALB/c mice treated with different Ang II-receptor blockers, corresponding to Figure 3F, (ns, no significance; **, P < 0.01).


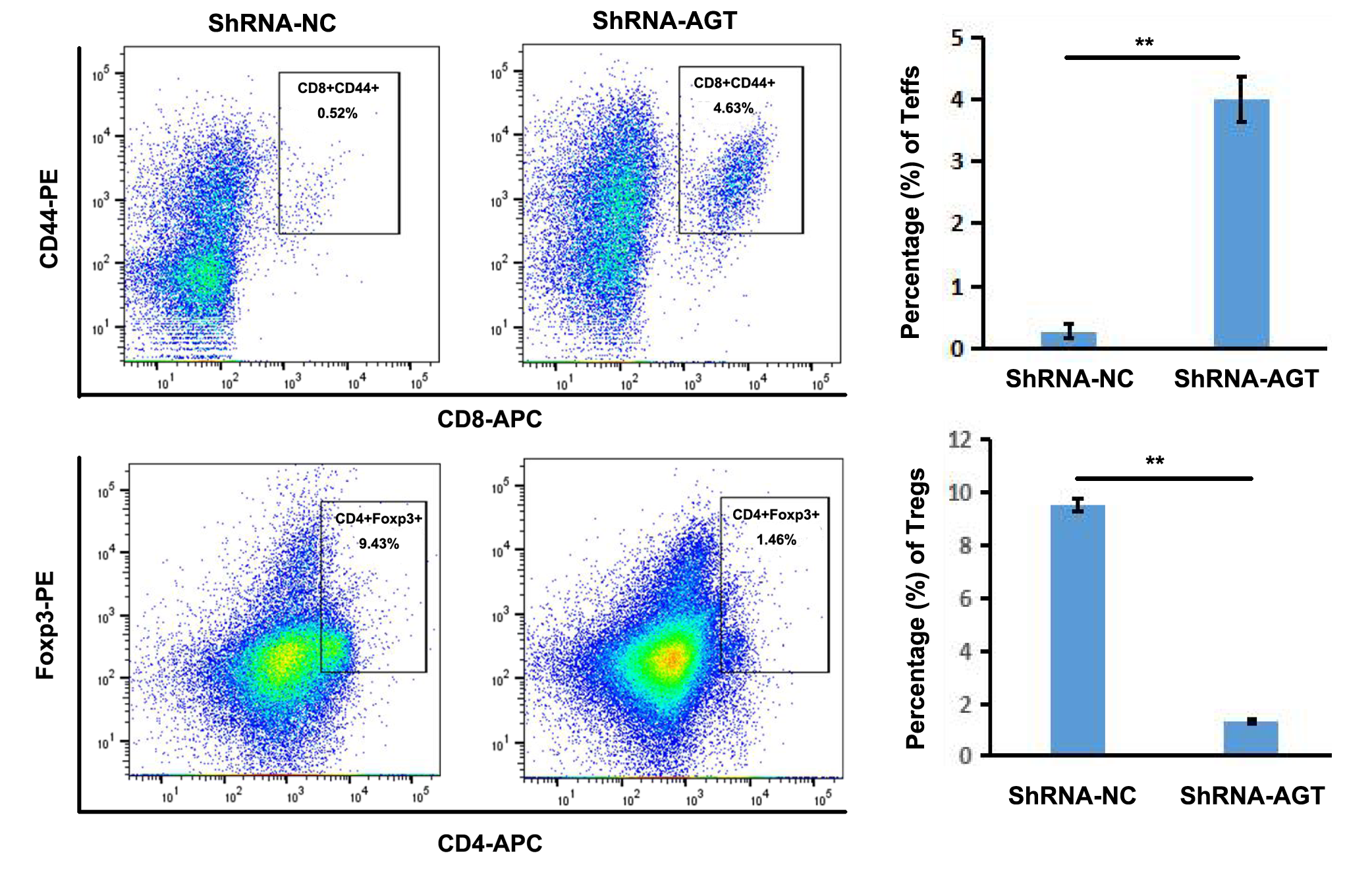


**Figure S6 AGT suppression reverses immunosuppressive tumor microenvironment.** Representative FACS plot of T_eff_ (CD8^+^CD44^+^) and T_reg_ (CD4^+^Foxp3^+^) in AGT-silenced and control 4T1 tumors from BALB/c mice. Bar chart (right) indicated statistic difference (**, P < 0.01). Data are presented as mean ± SEM, n=3.


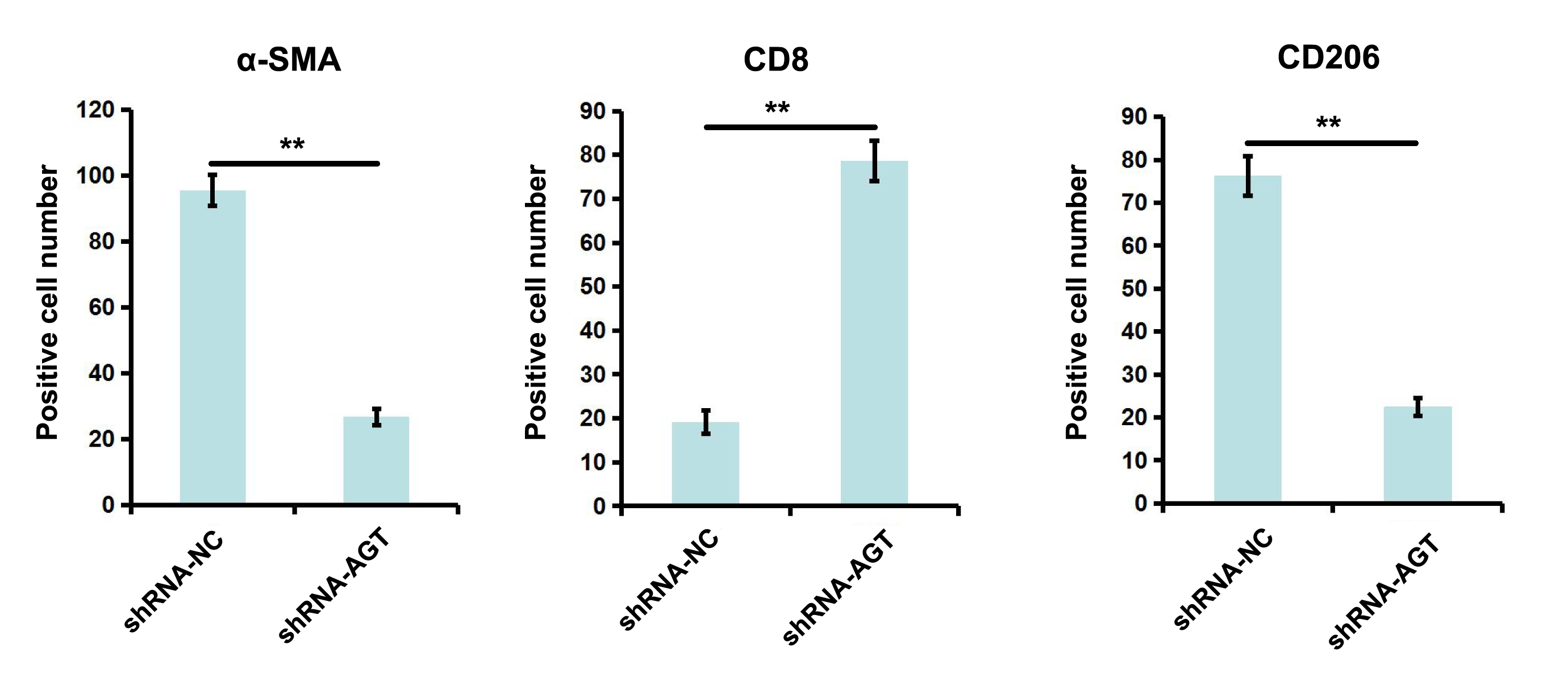


**Figure S7 α-SMA, CD8 or CD206 positive cells in** **pimonidazole-positive hypoxic regions of 4T1 tumors.** Positive cells were counted in 4 random 400× microscope visions in pimonidazole-positive hypoxic regions of AGT-silenced or control 4T1 tumors which were from 3 independent mice. (n=12, **, P < 0.01)


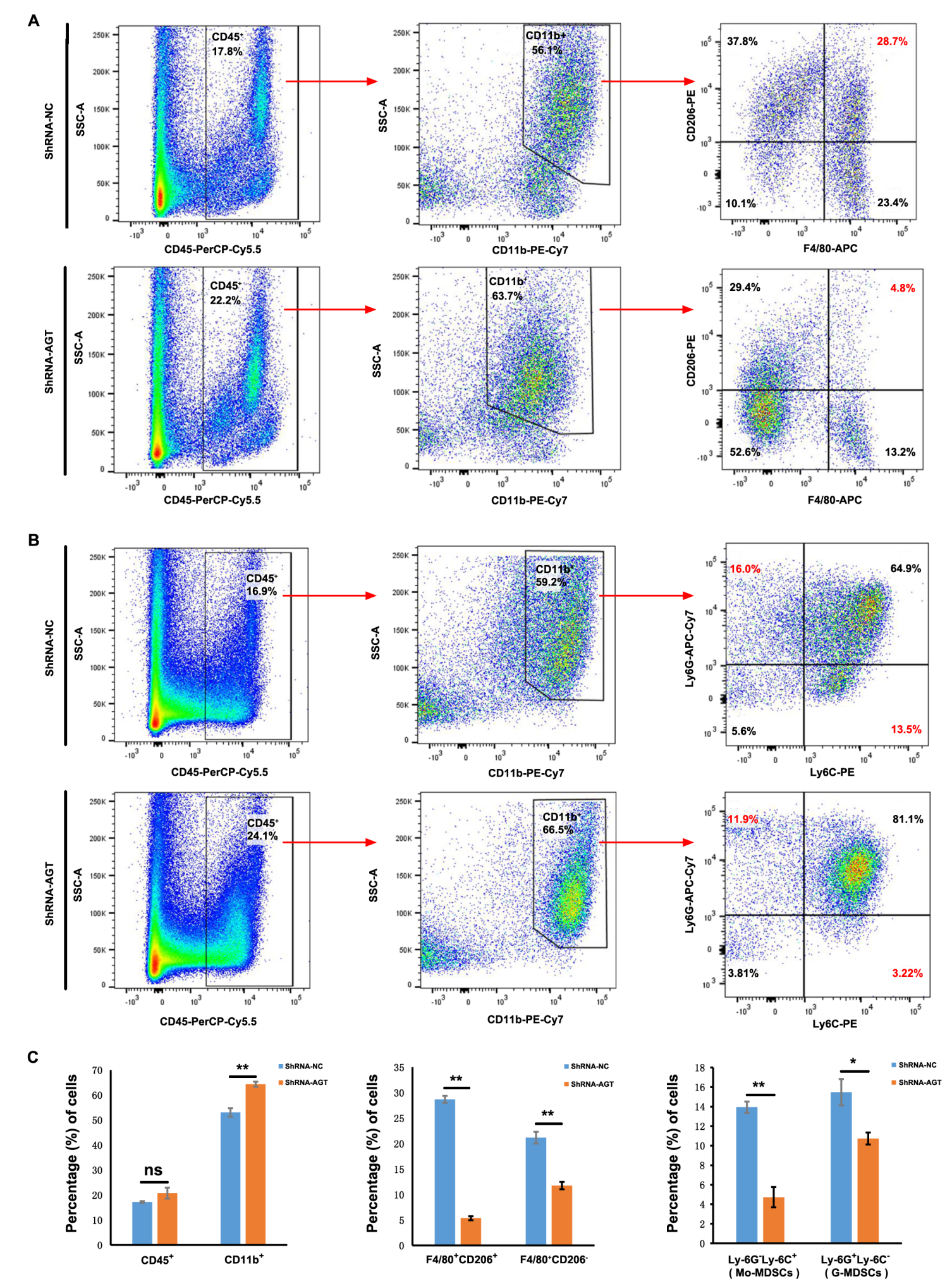


**Figure S8 The content of TAMs, Mo-MDSCs and G-MDSCs in shRNA-AGT and shRNA-NC 4T1 tumors.** (A) Representative FACS plot of TAMs (CD45^+^CD11b^+^F4/80^+^CD206^+^) in shRNA-AGT and shRNA-NC 4T1 tumors. (B) Representative FACS plot of Mo-MDSCs (CD45^+^CD11b^+^Ly6G^low^Ly6C^high^) and G-MDSCs (CD45^+^CD11b^+^Ly6G^high^Ly6C^low^) in shRNA-AGT and shRNA-NC 4T1 tumors. (C) Bar chart indicated the percentages of CD45^+^, CD11b^+^, TAMs, Mo-MDSCs and G-MDSCs in shRNA-AGT and shRNA-NC 4T1 tumors (ns, no significance; **, P < 0.01). Data are presented as mean ± SEM, n=3.

**A**


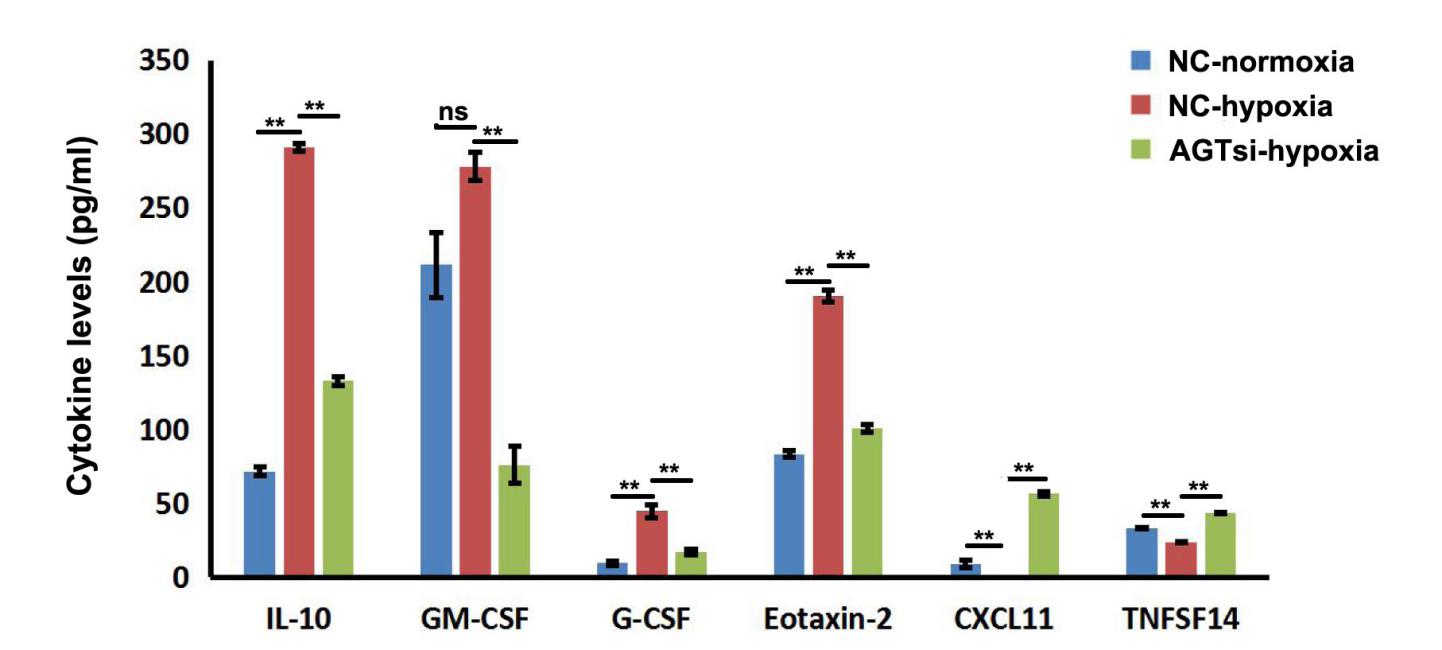


**B**


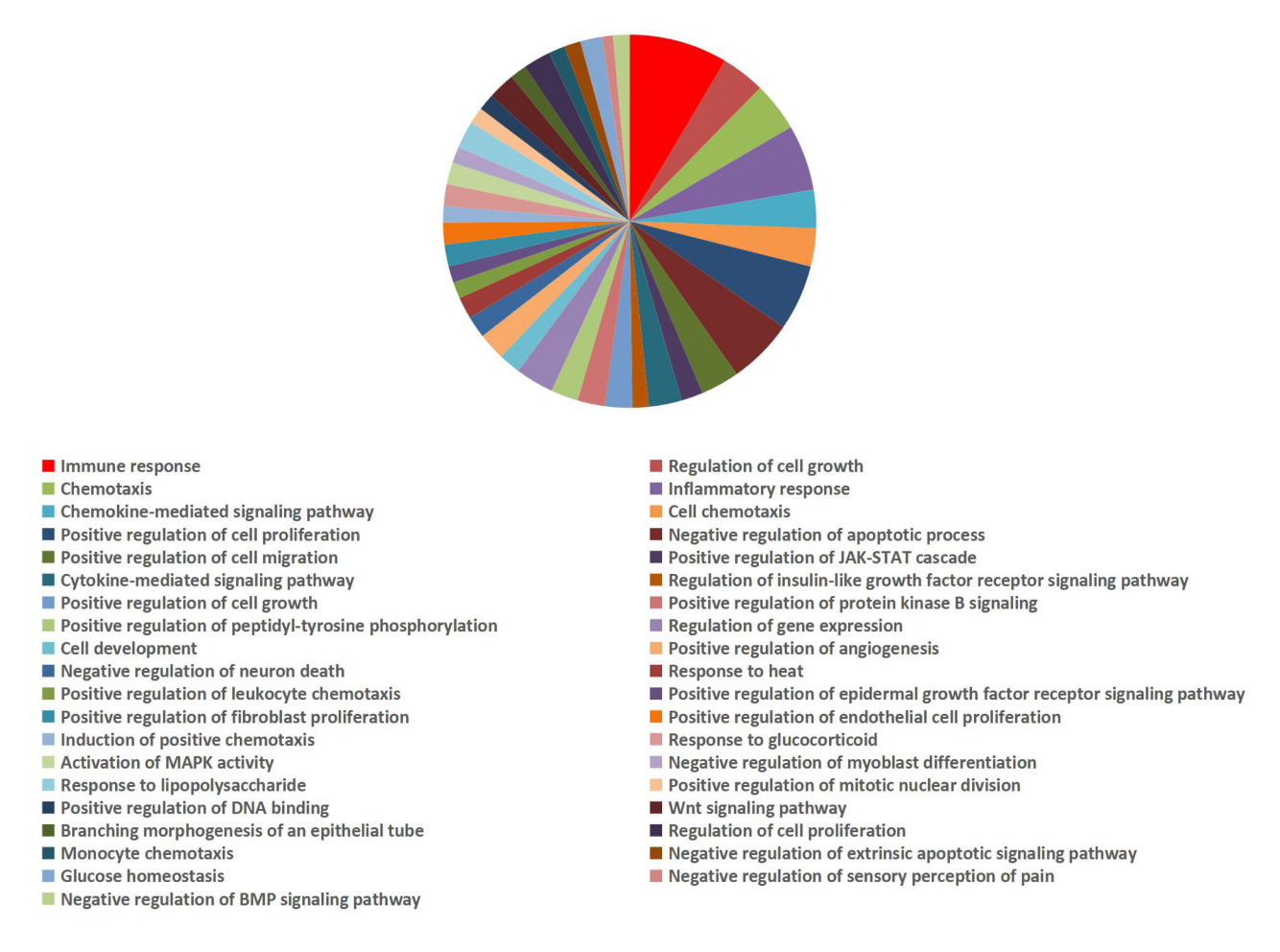


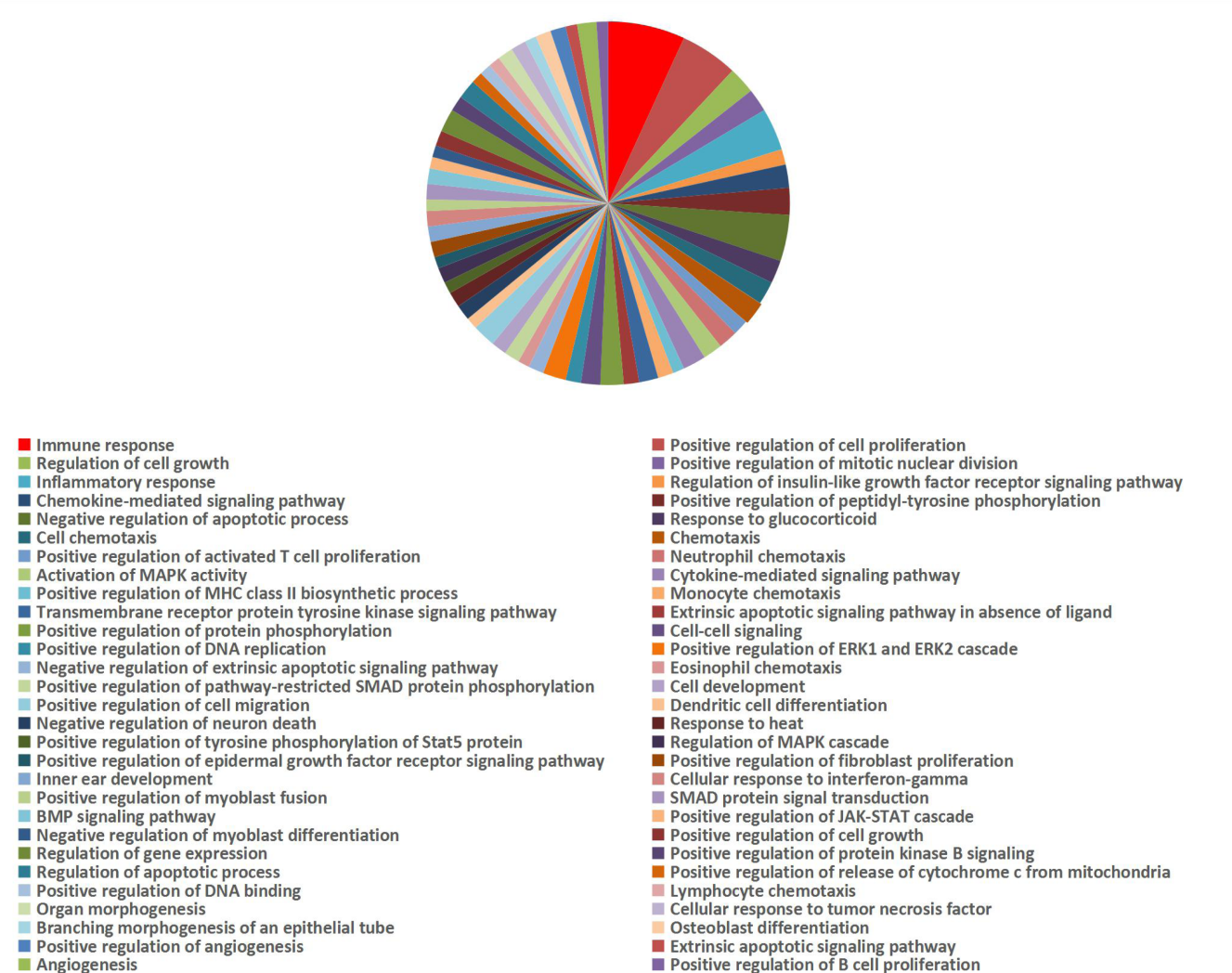


**C**

**D**


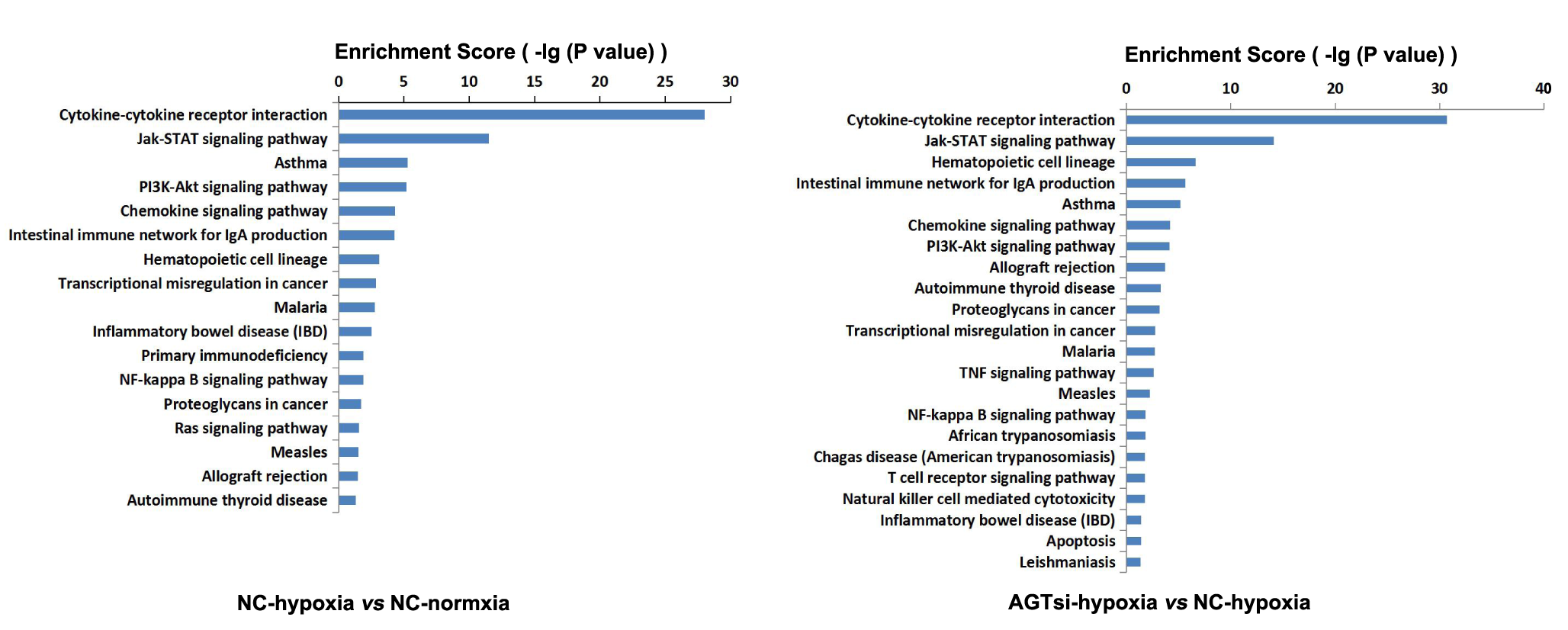


**Figure S9 AGT expression silencing triggers an immune-activating cytokine profile in hypoxic 4T1 cells. (A)** Detection of IL-10, GM-CSF, G-CSF, Eotaxin-2, CXCL11, and TNFSF14 levels in culture supernatant of 4T1 cells with AGT silence (AGTsi) or negative control (NC) under normoxia or hypoxia conditions, by ELISA analysis, verified the results from cytokine array (ns, no significance; **P < 0.01). (B) Gene Ontology (GO) analysis showed that hypoxia induced significantly higher frequencies of cytokines which were associated with 39 biological processes (p<0.01). (C) The cytokines influenced by AGT-silencing in hypoxia condition were associated with 58 biological processes (p<0.01). (D) Hypoxia influenced cytokines involving in 17 signaling pathways (left, p<0.05) and AGT silence affected cytokines involving in 22 signaling pathways (right, p<0.05).

**Supplementary Table**

**Table S1 Antibodies for Immunofluorescence**

| **Antigen** | **Cat. No.** | **Supplier** |
| --- | --- | --- |
| Rabbit anti-mouse alpha smooth muscle acting (α-SMA) | GTX100034 | GeneTex |
| Rat anti-mouse CD8 | Sc-18913 | Santa Cruz |
| Rabbit anti-mouse CD206 | Sc-58987 | Santa Cruz |
| Pimonidazole | HP2-1000Kit | Hypoxyprobe Inc. |
| rabbit anti-Angiotensin | 254552 | Abbiotec |
